# EST-Microsatellite Types and Structural Scenarios in European Hake Fisheries

**DOI:** 10.3390/ani12111462

**Published:** 2022-06-04

**Authors:** Alfonso Pita, María Fernández-Míguez, Pablo Presa

**Affiliations:** Laboratory of Marine Genetic Resources (ReXenMar), Centro de Investigación Mariña, Universidade de Vigo, 36310 Vigo, Spain; alpita@uvigo.es (A.P.); mariafernandezm@uvigo.es (M.F.-M.)

**Keywords:** APS, European hake, EST-microsatellite types, FAPS, fishery genetics, genetic structure, *Merluccius merluccius*, NAPS

## Abstract

**Simple Summary:**

The conservation of the maximum genetic background from all genomic regions matters for sustainability in the changing global scenario. This study focused the importance of integrating such genetic variation in order to infer meaningful units for management and sustainability. We show that knowledge that can be obtained from different marker types should be goal-oriented, i.e., remnant patterns of selection pressures and extreme drift episodes (directional markers, i.e., the evolutionary genetic background), current evolutionary novelty and adaptive potential for fisheries’ sustainability (balanced markers, i.e., the potential genetic drive of the species), and demographic dynamics of genetic relevance for fisheries’ management (neutral markers, i.e., the present-day fishery structure and connectivity). In particular, special attention should be paid to the variation in supergenes or balancing markers, which are a rich source of evolutionary novelty and can be crucial for species welfare and survival.

**Abstract:**

A fishery’s structure and connectivity are priors to its effective management. A successful description of such processes depends on both the sampling design and the choice of adequate genetic markers. EST markers are perfusing the studies of marine metapopulations and are believed to provide access to functional polymorphisms. However, the assumed adaptive role of outlier EST loci might not be generalizable. EST-microsatellites represent the upper polymorphic boundary in these regions because of their high mutation rate. We have subclassified the polymorphisms of EST-microsatellites to assess their structural contribution in the European hake, a paradigmatic and highly mobile marine species (HMMS). Because of the counterbalanced forces between directional markers (15%) and balanced markers (23%), the whole marker set offers the same structural situation as the one observed with neutral markers (62%), i.e., *k* = 2 gene pools. In contrast to outlier EST- microsatellites, neutral EST subsets allow one to measure crucial population phenomena for fisheries’ management. The high inter-population divergence of outlier EST-microsatellites is compatible with drifted post-selection genomic regions rather than with ongoing local selective pressures. The structural scenario in hake is explainable by a limited gene flow across the Almería-Oran Front (AOF) and by the within-basin IBD pattern of connectivity plus drift-related demographic events. This study highlights how polymorphic properties of EST-microsatellite types can be useful to address mutually excluding research tasks in fisheries, i.e., to address its evolutionary history (directional markers or FAPS: Fossil Adaptive Polymorphic Systems); to delineate management units (neutral markers or NAPS: Non Adaptive Polymorphic Systems); or to ensure sustainability (balanced markers or APS: Adaptive Polymorphic Systems).

## 1. Introduction

The long-term sustainability of fisheries demands a holistic knowledge combined with flexible and integrative models. Fishing decisions based upon such models should be able to identify the best-suited exploitation strategy for management units. Such units can be delineated based on diverse criteria (political, technical, administrative, etc.), although they are often incongruent with ecological evidence [1]. A realistic delineation of management units requires a significant knowledge of their biological properties (trophic relationships, reproductive determinants, etc.), as well as their spatiotemporal genetic patterns [2] at three levels of complexity, i.e., within cohorts or among lifecycle transitions [3], within species or among regional conspecific stocks [4], and between species [5]. The spatiotemporal genetic connectivity between those organizational levels is a multi-dimensional vector network where the evolutionary dynamics of species progress. Assuming that nothing in evolution makes sense without connectivity, e.g., between gametes, between individuals, or between species, its intensity determines our capacity to identify spatiotemporal genetic structures within metapopulations. Connectivity and genetic structuring are inverse processes [6] and priors to optimal management because they are responsible for the maintenance of the metapopulation pattern of a given species. Despite such importance, they are largely unknown in most fisheries (in terms of the stability of life cycles, mesoscale and macro-oceanic fish circuits, stability and intensity of migration patterns, causality of the genetic divergence between demes, etc.). This lack of knowledge is believed to be responsible for our inability to venture increases and collapses in fish stocks [7]. In practice, the ability to model connectivity patterns and genetic structures in fisheries depends on the sampling design as well as on the choice of genetic tools fitting the resolution level of the fishery structure we are trying to solve. An incorrect choice of the genetic marker in type or number can lead to diagnosing either panmixia or structuring of the same fishery [8].

Specific strategies aimed at measuring life cycle connectivity in metapopulations, such as cohort analyses [9], mark/recapture [10], otolith biochemistry [11], and genetic markers, offer useful but partial information on fish dynamics. This is due to the lack of spatial or temporal sensitivity of the tools to changes in connectivity during the life cycle [5]. Therefore, integrative analyses of biochemical tags of different imprints and evolutionary paces with georeferenced tags are a necessary approach to model more realistic vector networks of connectivity in fisheries [12]. Among genetic markers, microsatellites have been the most widely applied tools to investigate the genetic structure of fishes in the last two decades [13] in terms of their being endowed with neutral and quasi-neutral polymorphisms [14] and being appropriate to solve fine-scale relationships [15]. More recently, Expressed Sequence Tags (ESTs) from transcriptomic profiles (and SNPs therein) [16] are perfusing population studies as they are believed to reflect local adaptation and cryptic population structures [17]. Theoretically, outlier ESTs could respond to ongoing directional selection, but there are reasonable doubts about such generalization [18] as well as on their suitability to measure both population dynamics and real fishery structures [19]. Moreover, punctual genotyping efforts provide punctual assessments of population structures that are interannually fluctuating in highly mobile marine species (HMMS) [20].

The European hake *Merluccius merluccius* is a paradigmatic demersal fish from the NE Atlantic on which large discrepancy has been reported regarding the microstructure depicted with SNPs from ESTs (e.g., *k* = 4 gene pools, [21]) and the highly connective unstructured Atlantic metapopulation (except the restricted gene flow with the North Sea) as depicted with both microsatellites [22] and SNPs [23]. A similar divergent insight can be found on the Mediterranean hake, where a multi-stock scenario (*k* = 3) was proposed on a few outlier SNPs [24] as opposed to the single Mediterranean metapopulation suggested in previous studies with several marker types [22,25]. Otherwise, there is a general consensus on the structuring of major species into an Atlantic subpopulation and a Mediterranean subpopulation [26]. Such a major divide is thought to be mediated by the Almería–Oran Oceanographic Front (AOF, [27]), a persistent oceanographic barrier to gene flow in many marine taxa [28]. However, the causal role of AOF on the parapatric divergence of hake has been overshadowed by basin-dependent adaptive hypotheses worked out on outlier ESTs [29]. While neutral microsatellites are of little use to disclose adaptive differences between groups [30], EST-microsatellites represent the upper polymorphic boundary of allele diversity in those functional regions because of their high mutation rate. This property would allow for both assessing the contribution of neutral and non-neutral variation in depicting the divergence between basins or among regions in hake and to help clarifying the opposite structural scenarios reported so far.

In this study, we dissected EST-microsatellites into marker types to assess their structural information on European hake fisheries. The recognized marker types were (a) unrestricted non-functional polymorphisms (neutral markers or NAPS: Non Adaptive Polymorphic Systems) whose range distributions were only shaped by geographic and demographic phenomena, (b) purified polymorphisms (directional markers or FAPS: Fossil Adaptive Polymorphic Systems) shaped by historical evolutionary events, and (c) stabilizing polymorphisms (balanced markers or APS: Adaptive Polymorphic Systems) from genomic regions leading the adaptive drive of the species. Our general working hypothesis states that EST-microsatellites might exhibit an outlier divergence between basins due to past selective forces producing a deflated polymorphism, which is nowadays shaped by connectivity determinants. Therefore, their outlier status would be caused by both the inter-basin phylogeographic barrier and the demographic intra-basin IBD pattern. It is expected that the population scenarios afforded from each marker type would give clues on their usefulness to pursue goal-oriented tasks in research on fisheries.

## 2. Materials and Methods

### 2.1. Development of EST-Microsatellites

EST-microsatellites were developed from published entries of a European hake muscle transcriptome [31] performed with the sequencing platform Roche 454 FLX (http://www.ebi.ac.uk/ena/data/view/ERP000950, accessed on 22 May 2020). Tandem repeat regions were selected from the EST library following the roadmap: sequence cleaning, microsatellite detection, sequence redundancy, selection of microsatellites, and primer design (Figure S1). All those steps followed indications of the open access DNA analytical package QDD2 [32]. Sequences fulfilling the most stringent conditions enforced in QDD2, i.e., tandem repeat purity, suitability for PCR amplification, and optimal primer pair design, were classified according to their amplicon size. Large di-, tri-, and tetranucleotide tandem repeats were prioritized for their higher expected polymorphism [33]. Potential markers were tetraplexed upon both the similarity of their annealing temperature and the non-overlapping amplicon sizes. Multiplexes were tested in silico using the web tool FastPCR [34] aimed to check for primer dimers within and between markers as well as to identify putative multiple complementary DNA targets. FastPCR was used to assess the expected quality of multiplex PCR amplifications and the amplicons’ size. Real single-locus PCR amplifications were performed before multiplexing in order to optimize the amplification conditions. Renewed multiplexed marker combinations were enforced to achieve the optimal co-amplification of all candidate tetraplexes. Fluorophores 6-FAM and HEX were used to label the forward primer of each marker upon the amplicon size for compatibility with allele scoring. PCR products were visualized in 2.5% agarose gels (CSL-AG500, Cleaver Scientific Ltd., Rugby, UK), and markers showing a poor amplification quality were discarded. All the selected microsatellite-containing sequences were compared against the National Center for Biotechnology Information (NCBI) database on 22 May 2020 using the tool BLASTX 2.10.1 [35] to check for molecular homology with known homologous or heterologous proteins of fish species.

### 2.2. Sampling, DNA Extraction, and Microsatellite Genotyping

**Figure 1:**
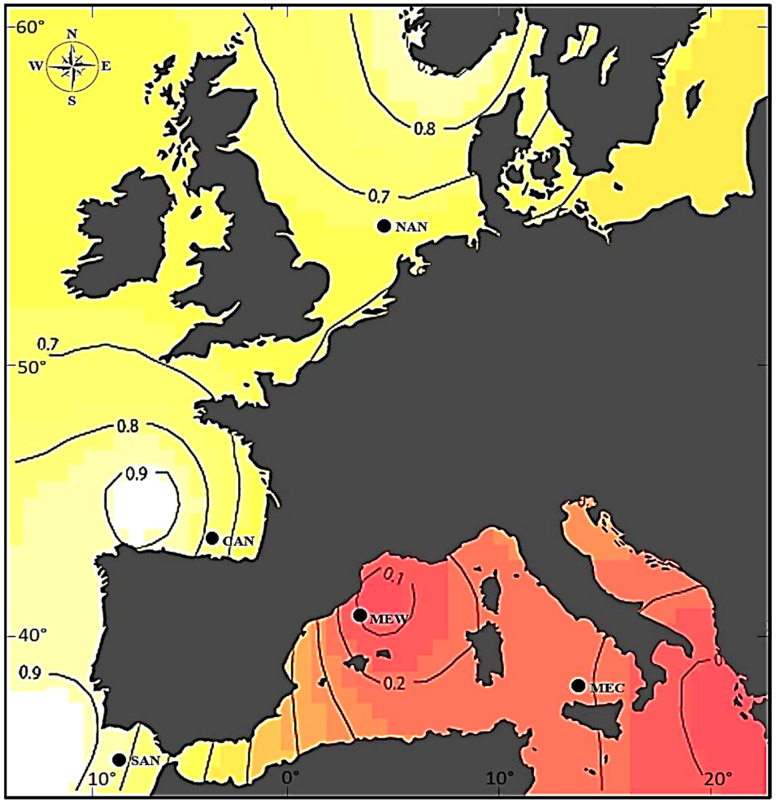
Posterior probability of ascription of sampled regions to one of the two subpopulations of *Merluccius merluccius*, one in the Atlantic North (NAN, CAN, SAN) and the other in the Mediterranean Sea (MEW, MEC). The Bayesian inference was computed with GENELAND on multilocus data from 26 microsatellites. Regions: NAN, North Sea, 55°30′ N 04°36′ E (n = 31); CAN, Cantabrian Sea (from Gijón to Labra de Laredo), 43°32′ N 03°26′ W (n = 40); SAN, Gulf of Cádiz (from Sardaos to Barra de Huelva), 36°21′ N 07°06′ W (n = 40); MEW, West Mediterranean Sea (from Tarragona to Balearic Islands), 41°02′ N 02°33′ E (n = 40); MEC, Central Mediterranean Sea (from Sardinia to Castellammare), 38°03′ N 12°56′ E (n = 41).

Five major regions of the European hake were sampled onboard commercial ships and research vessels in the Atlantic Ocean and the Mediterranean Sea in August 2019 (Figure 1). A piece of muscle from each individual was preserved in 96% ethanol until the extraction and purification of genomic DNA following the FENOSALT method [36]. The samples were genotyped with microsatellites from two sources: (1) five a priori neutral microsatellites (*Mmer*-hk3b, *Mmer*-hk9b, *Mmer*-hk20, *Mmer*-hk29, and *Mmer*-hk34b [37]), as labeled, multiplexed, and amplified using conditions described in [38], and (2) twenty-one a priori non-neutral microsatellites (this study) as characterized from a muscle transcriptome of hake [31]. Multiplexed reactions of 15 μL contained ≈ 40 ng template DNA, 300 µM per dNTP, 0.3 µM per primer and 1.25 U of Taq DNA polymerase (BIOLINE). The amplification routines were carried out in a Mastercycler Gradient Thermal Cycler (EPPENDORF) at the specific annealing temperature and MgCl_2_ concentration. Cycling conditions consisted of an initial denaturation step at 95 °C for 5 min followed by 35 cycles of denaturation at 95 °C for 70 s, annealing at the locus-specific temperature for 70 s and extension at 72 °C for 80 s. A final elongation step was carried out at 72 °C for 30 min. Fragment separation of labeled PCR products was performed in an ABI PRISM 3130 Capillary Sequencer (CACTI—Scientific and Technological Research Assistance Centre, University of Vigo) using GeneScan 500 ROX internal-lane size ladder as a reference marker for allele size determination. Allele scoring was performed using GeneMarker v2.4.2 (SOFTGENETICS), and genotyping errors were minimized by comparison of simultaneous readings from independent researchers as well as re-genotyping dubious or negative amplifications.

### 2.3. Selection Signature and Power Testing

Putative selection on sequence polymorphisms was explored on two datasets, i.e., 5 anonymous microsatellites and 21 EST-microsatellites, under two population scenarios, i.e., between basins (the Atlantic Ocean and the Mediterranean Sea) as well as among five regions from most of the species range (Figure 1). The first approach aimed to infer directional selection on microsatellite polymorphisms was that implemented in LOSITAN [39] which uses the *F*_ST_-based outlier method (fdist [40]). This method allows the detection outlier loci with a significant *F*_ST_ departure from neutral expectations. Correction for multiple tests was performed with the False Discovery Rate method (FDR) [41]. A second method to infer stabilizing or balancing selection was that implemented in BAYESCAN v2.1 [42], which uses differences in allele frequencies among populations. A total of 550,000 iterations (sample size = 50,000; thinning interval = 10; burn-in = 50,000) were set to run the Reversible Jump Markov Chain Monte Carlo algorithm (RJ-MCMC) implemented in BAYESCAN. That treatment allowed for the estimation of posterior probabilities of including/excluding the alpha component under selection, where alpha is the locus-specific component of the *F*_ST_ shared by all subpopulations, so one can assume a specific locus departure from neutrality when alpha differs significantly from zero. In order to adjust the proposal distributions for the RJ-MCMC, twenty pilot runs of 50,000 iterations were each set off before starting calculations. The prior odds settings for the neutral model were 10, as recommended to, reduce false selection positives [42]. Outlier loci were identified after the *q*-value upon the FDR test and the Jeffreys’s scale of evidence for Bayes Factors (BF) using Posterior Odds (PO) instead of BF. The *F*_ST_-values obtained with BAYESCAN and LOSITAN were compared using a statistical regression implemented in IBM SPSS Statistics v22. Power to falsify the null hypothesis of genetic homogeneity among subpopulations was tested for all loci combinations using POWSIM v4.1 [43] assuming an effective population size of 1000 with 1000 replicates of the entire process (drift, sampling, and statistical testing). The number of generations simulated by POWSIM over a specific base population varied in order to yield the same *F*_ST_-value observed among subpopulations. Power estimates were obtained after the chi-square test of Pearson as implemented in POWSIM. The proportion of false significant tests (type I error, *p* < 0.05) was calculated with the above routine but omitting the drift steps (i.e., *F*_ST_ = 0).

### 2.4. Gene Diversity and Subpopulation Structure

The number of alleles, allele frequencies, and allelic richness per subpopulation and per basin were calculated with FSTAT v2.9.3.2 [44]. Differences in the mean number of alleles between pairs of marker types (neutral, directional, and balancing) were explored with a Mann–Whitney test from XLSTAT. Observed heterozygosity (*H*_O_), expected heterozygosity (*H*_E_), and Hardy–Weinberg Equilibrium tests (HWE) were carried out with GENEPOP v4.0 [45]. The fixation index within subpopulations (*F*_IS_) and its associated probability of differing from zero were calculated using 20 batches and 5000 iterations per batch of the Markov Chain Method implemented in GENEPOP v4.0. Genotypic linkage disequilibrium tests for all pairs of loci and all possible scenarios were performed in GENEPOP v4.0 using the following Markov chain parameters: 10,000 dememorization steps, 100 batches, and 5000 iterations per batch. The Analysis of Molecular Variance [46] as implemented in ARLEQUIN v3.5.1.2 [47] was used to calculate the molecular variance between basins as well as among regions within basin upon types of microsatellites and the types of selection, i.e., LOSITAN (directional selection) and BAYESCAN (balancing selection). Hierarchical AMOVA was also computed on 16 and 21 neutral microsatellites, among regions and between basins, respectively. The number of gene pools (*k*) in the dataset was estimated using 1 × 10^6^ Bayesian iterations (Atlantic–Mediterranean scenario) and 2 × 10^6^ Bayesian iterations (five-regions scenario) under the uncorrelated allele frequency model [48] and the spatial model [49] as implemented in GENELAND v3.2.4 [50]. The *k*-parameter was also explored under spatial population analyses considering a mixture model [51] using 100,000 iterations of the admixture model [52] as implemented in BAPS v5.2 [53]. The regression of the genetic distance (*F*_ST_/(1 − *F*_ST_)) and the geographical distance between sampling locations (following the continental shelf edge) was explored with the Mantel permutation procedure implemented in ISOLDE from GENEPOP 4.0.

## 3. Results

### 3.1. Development of EST-Microsatellites

The 0.5 M EST reads screened from a European hake cDNA library produced 17,655 microsatellite-containing sequences, among which primer design was feasible on 1483 sequences (Figure S1). One hundred ninety sequences contained pure microsatellites (2–6 bp motif ≥ 6 repeats), lacked homopolymers and nanosatellites in the flanking regions, and showed the lowest penalty on primer pairs. Forty-eight microsatellites were theoretically compatible with each other for PCR multiplexing in 12 tetraplexes. Twelve real single-locus amplifications showed suboptimal amplifications, and thirteen loci exhibited no polymorphism. The remaining 23 polymorphic EST-microsatellites were rearranged in multiplexes and assayed in equimolecular mixtures until reaching their optimal coamplification. The final EST microsatellite panel was formed by 9 di-, 12 tri-, and 2 tetra-nucleotide repeats combined in one triplex (Triplex#2) and five tetraplexes (Tetraplex#1–5) (Table S1). Markers *Mmer*-EST_3.1 and *Mmer*-EST_2.2 were removed from further analyses due to their uneven amplification across individuals. Five anonymous di-nucleotide microsatellites were PCR-amplified in two simplex and one triplex reactions (Triplex 1, Table S1).

### 3.2. Genetic Diversity in Natural Samples

All microsatellite markers were polymorphic, ranging from 6 alleles of locus *Mmer*-EST_9.1 in the Mediterranean to 34 alleles of locus *Mmer*-EST_8.4 in the Atlantic (Table S1). Diversity parameters did not differ between microsatellite sources, i.e., random cloning (N_a_ = 18.40 ± 6.72; *H*_O_ = 0.64 ± 0.17; *H*_E_ = 0.88 ± 0.08) versus EST-microsatellites (N_a_ = 17.55 ± 8.00; *H*_O_ = 0.64 ± 0.15, *H*_E_ = 0.85 ± 0.10). Significant deviations from HWE were observed in 25 loci. Adjustment to HWE was observed in 6 out of 26 loci in the Atlantic (*Mmer*-hk3b, *Mmer*-EST_1.1, *Mmer*-EST_13.1, *Mmer*-EST_10.2, *Mmer*-EST_13.3, *Mmer*-EST_8.1) and in 2 out of 26 loci in the Mediterranean (*Mmer*-EST_10.2, Mmer-EST_8.2) (Table S1). Linkage disequilibrium was observed in 16 out of 325 pairwise tests (4.92%) in the Atlantic and in 18 out of 325 tests (5.54%) in the Mediterranean. At the basin level, the polymorphism of four balanced loci (N_a_ = 24.38 ± 5.88) was significantly higher than that of 21 neutral loci (N_a_ = 16.79 ± 7.49; Mann–Whitney U-test = 266.5, two-tailed *p* = 0.009) as well as that of one directional locus (N_a_ = 10.50 ± 0.71; Mann–Whitney U-test = 16.00, two-tailed *p* = 0.049). The two latter categories did not differ from each other (Mann–Whitney U-test = 63.5, two-tailed *p* = 0.550). At the five-region level, the polymorphism of 6 balanced loci (N_a_ = 19.40 ± 4.00) was significantly higher than that of 17 neutral loci (N_a_ = 12.08 ± 4.89; Mann–Whitney U-test = 1840, two-tailed *p* < 0.0001) and that of directional loci (N_a_ = 8.33 ± 2.97; Mann–Whitney U-test = 369.50, two-tailed *p* < 0.0001). The two latter categories also differed significantly from each other (Mann–Whitney U-test = 921, two-tailed *p* = 0.006).

### 3.3. Functional Annotation, Selection Signature, and Power Testing

None of the anonymous microsatellite sequences, as well as 86% (18/21) EST sequences matched proteins from databases. Fourteen percent of EST sequences (3/21) containing microsatellites (*Mmer*-EST_10.1, *Mmer*-EST_8.1 and *Mmer*-EST_8.2) showed significant matches with five proteins and one coenzyme from 75 fish species (*E*-value cut-off threshold ≤ 1.00 × 10^−4^), i.e., transmembrane emp24 protein 3, transmembrane emp24 protein 7, Isocitrate dehydrogenase, MTSS1, MTSS2, and protein scribble homolog (Table S2). None of those protein-matched sequences showed signatures of selection upon *F*_ST_ values. The fdist *F*_ST_ outlier method of LOSITAN identified locus *Mmer*-hk3b as a candidate for directional selection in both population scenarios (*p* = 0.999, Table 1), while the other anonymous loci ranked neutral. Three EST sequences (*Mmer*-EST_9.1, *Mmer*-EST_14.2 and *Mmer*-EST_6.4) were candidates for diversifying selection in the five-regions scenario, and none of them were between Atlantic and Mediterranean basins (Table 1). The algorithm implemented in BAYESCAN classified loci *Mmer*-hk34b, *Mmer*-hk9b, and *Mmer*-hk20 as candidates for balancing selection in both population scenarios, while loci *Mmer*-hk3b and *Mmer*-hk29 ranked neutral (Table 1). Sequence *Mmer*-EST_6.3 was candidate to balancing selection between basins as well as among regions. Loci *Mmer*-EST_13.3 and *Mmer*-EST_11.2 were balanced candidates among regions. None of the outlier loci matched known proteins from databases.

**Figure 2:**
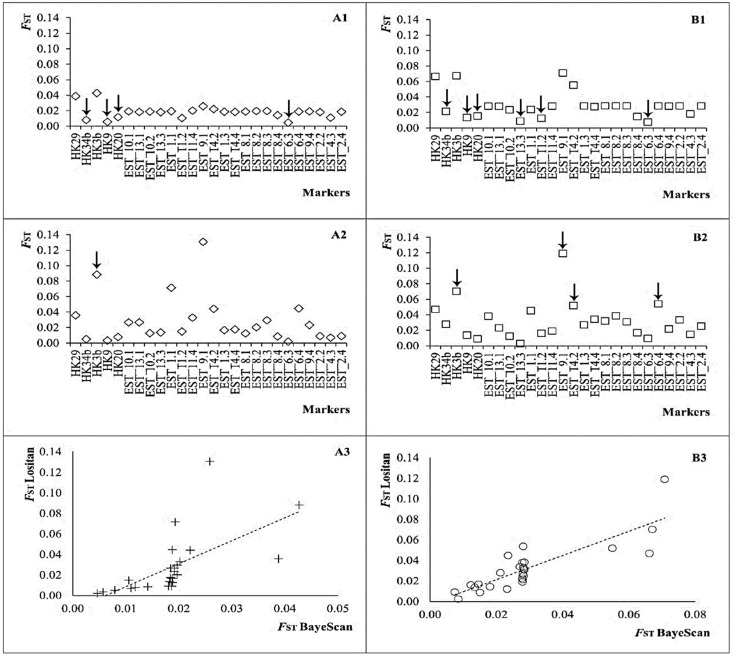
Interpopulation fixation index *F*_ST_ per microsatellite calculated between Atlantic and Mediterranean hake subpopulations (panel (**A**): (**A1**), *F*_ST_ from BAYESCAN; (**A2**), *F*_ST_ from LOSITAN; (**A3**), correlation between *F*_ST_ estimated with BAYESCAN and LOSITAN (*Y =* 2.241*X* − 0.0138; *R*^2^ *=* 0.400; *F =* 16.006; *p =* 0.001)) and among five regions (panel (**B**): (**B1**), *F*_ST_ from BAYESCAN; (**B2**), *F*_ST_ from LOSITAN; (**B3**), correlation between *F*_ST_ estimated with BAYESCAN and LOSITAN (*Y =* 1.1806*X* − 0.0024; *R*^2^ *=* 0.714; *F =* 59.755; *p <* 0.001)). Arrows indicate loci with significant departure from neutrality (see Table 1).

All candidate loci under balancing selection after BAYESCAN ranked neutral in LOSITAN and all those directional loci after LOSITAN ranked neutral in BAYESCAN (Figure 2). The statistical regression between method-dependent *F*_ST_ measures was significant in the two population scenarios assessed (Figure 2). BAYESCAN *F*_ST_-values per locus between basins ranged from 0.005 to 0.043 (*Mmer*-EST_6.3 and *Mmer*-hk3b, respectively) and LOSITAN *F*_ST_-values ranged from 0.002 to 0.131 (*Mmer*-EST_6.3 and *Mmer*-EST_9.1, respectively) (Table 1; Figure 2). BAYESCAN *F*_ST_-values per locus among regions ranged from 0.008 to 0.075 (*Mmer*-EST_6.3 and *Mmer*-EST_9.1, respectively) and LOSITAN *F*_ST_-values ranged from 0.002 to 0.119 (*Mmer*-EST_13.3 and *Mmer*-EST_9.1, respectively) (Table 1; Figure 2).

The statistical power of most markers (20 EST-microsatellites and 5 anonymous microsatellites), as well as that of their functional subsets, was 1.0 irrespective of the hierarchical partition enforced (five regions or two basins) under simulated *F*_ST_ = 0.0252 and *F*_ST_ = 0.0247, respectively. Using the whole dataset, type I error was 0.026 (five regions) and 0.033 (two basins) (Table S3). Locus *Mmer*-EST_6.3 lacked statistical power to refute the null hypothesis. The single directional locus (*Mmer*-hk3b) showed power >0.99 to reject the null hypothesis of the Atlantic–Mediterranean genetic homogeneity.

### 3.4. Structural Population Genetics

The Bayesian algorithms of BAPS and GENELAND as applied to functional microsatellite subsets provided a *k* range of 1–2 gene pools in the two-basins scenario and a *k* range of 1–3 gene pools in the five-regions scenario (Table S4). The subsets of 1 to 4 directional loci after LOSITAN provided an invariable *k* = 2 across hierarchical levels, except a single *k* = 3 among regions with GENELAND (Table S4). The subsets of four and six balanced loci after BAYESCAN provided an invariable *k* = 1 except an aberrant *k* = 2 among regions. The subsets of 22 and 20 neutral loci after BAYESCAN provided invariable both, *k* = 1 after BAPS and *k* = 2 after GENELAND. Both Bayesian methods recovered a *k* = 1 scenario across hierarchical levels from balanced markers but *k* =2 from neutral markers upon BAYESCAN and comprising directional markers upon LOSITAN (Table S4). Consensus *k* = 1 and *k* = 2 were retrieved by BAPS and GENELAND, respectively, across hierarchical levels (two basins, five regions) either using the whole set of 26 markers or using the subsets of neutral markers per hierarchical level, i.e., 21 loci between basins and 16 loci among regions (Table S4; Figure 2).

The genetic variance as broken down per region provided a significant inter-basin divergence (*F*_CT_ = 0.018, *p* ≤ 0.010) upon anonymous microsatellites and EST-microsatellites or their combination (Table S5). The genetic variance as broken down per basin increased to *F*_CT_ = 0.026 (*p* ≤ 0.010) (Table 2). The single directional locus between basins after LOSITAN (*Mmer*-hk3b) exhibited more than three-fold higher divergence (*F*_CT_ = 0.091, *p* ≤ 0.010) than 25 neutral loci (*F*_CT_ = 0.024, *p* ≤ 0.010). BAYESCAN did not show differentiation between basins when variance was broken down either per region (six balanced loci, *F*_CT_ = 0.003, *p* = 0.183) (Table S6) or per basin (four balanced loci, *F*_CT_ = 0.005, *p* = 0.016) (Table 2). The remaining 22 “neutral” loci subset that comprised the directional locus of LOSITAN was shown to be significant (*F*_CT_ = 0.031, *p* ≤ 0.010) (Table 2). After the marker set with the two classification workbenches was filtered, the two subsets of absolute neutral loci produced a significant divergence between basins (21 loci, *F*_CT_ = 0.028, *p* ≤ 0.010), as well as among regions (16 loci, *F*_SC_ = 0.020, *p* ≤ 0.010). The algorithm of BAPs produced *k* = 1 gene pools in the whole range, between basins as well as among regions, except when directional markers were involved. The algorithm of GENELAND produced *k* = 2 gene pools in the whole range, between basins as well as among regions, except when balanced markers were involved (Table 2).

**Figure 3:**
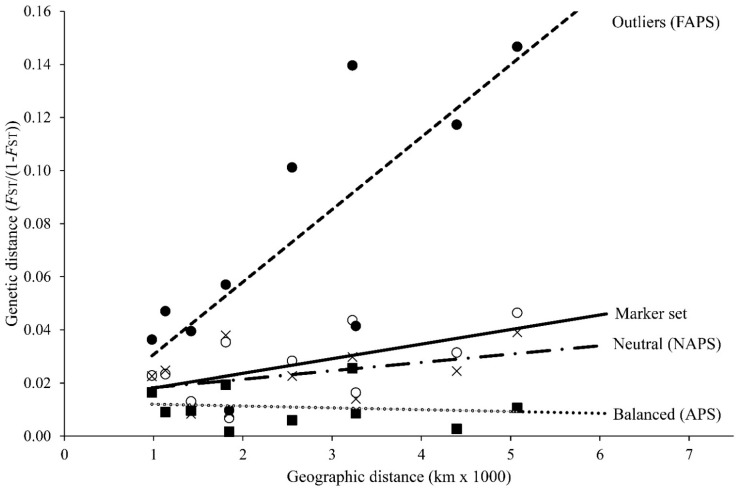
Pairwise test of isolation by distance among five regions of the European hake upon four marker types. Balanced: six balanced markers (closed squares and dotted line; *Y =* −7 × 10^−7^*X* + 0.0127; *R*^2^ = 0.0163; *p* = 0.641); Neutral: 16 absolute neutral markers (crosses and dashed-dotted line; *Y =* 3 × 10^−6^*X* + 0.0151; *R*^2^ = 0.1685; *p* = 0.193); Full marker set: 26 microsatellites (open circles and continuous line; *Y =* 5 × 10^−6^*X* + 0.0126; *R*^2^ = 0.3565; *p* = 0.064); Outliers: 4 outlier markers (closed circles and dashed line; *Y =* 3 × 10^−5^*X* + 0.0034; *R*^2^ = 0.6218; *p* = 0.020).

The isolation by distance pairwise test on regional samples was significant upon the outlier marker subset (*Y =* 3 × 10^−5^*X* + 0.0034; *R*^2^ = 0.6218; *p* = 0.020) and marginally significant upon the full marker set admixture *(Y =* 5 × 10^−6^*X* + 0.0126; *R*^2^ = 0.3565; *p* = 0.064). No significant IBD was observed with either strictly neutral markers or balanced markers (Figure 3).

## 4. Discussion

A paradigmatic phylogeographic divide is the one caused by the Almería–Oran Oceanic Front (AOF) in the westernmost side of the Alboran Sea [27]. This AOF has proven to be a temporal stable barrier to gene flow [55] in several species [56], including the European hake [57]. A population challenge in the European hake scenario is to clarify if such AOF is responsible for maintaining the inter-basin divergence or if basin-associated selective forces also add up to the AOF-mediated drift, e.g., [58]. The amount of genetic divergence between populations usually differs among studies upon the per-locus selective pressures embedded in the marker set applied. Those differences between marker sets generate conflicting views in hake, such as the quasi-panmictic metapopulation scenario depicted within basins with anonymous microsatellites [22] versus the ultrastructural scenarios reported on *F*_ST_ outliers from EST-SNPs (e.g., *k* = 6, [24]). Several questions of broader interest are dealt with herein using EST-microsatellites because of their expected high polymorphism within putative adaptive regions. For example, what is the cause of the inter-basin divergence? What are the genetic scenarios afforded from each marker type to address significant fishery structures? What marker-type diversity matters for sustainability?

### 4.1. The Polymorphic Properties of the Marker Set

The lack of differences between the polymorphism of EST-microsatellites and that from anonymous microsatellites differs from previous differences reported between those microsatellite sources [59]; i.e., as discussed later in this section, all outlier microsatellites exhibited significant lower polymorphism than the rest of microsatellite types. Additionally, the classification of markers per type upon selective constraints responded to the discriminant algorithm employed [60] but not to their cloning source; i.e., not all the anonymous microsatellites were neutral and not all the EST-microsatellites were non-neutral. Directional loci after LOSITAN were those exhibiting upwards outlier *F*_ST_ values, while all the rest were classified as neutral loci, i.e., no balancing selection was retrieved from LOSITAN. Conversely, balanced loci after BAYESCAN were those exhibiting downwards outlier *F*_ST_ values, while the rest were classified as neutral, including those with upwards *F*_ST_ outliers in LOSITAN. Consequently, both algorithms seem to be required when it comes to defining the precise per-locus selection status within a marker set [61].

The structural scenarios afforded between basins as well as within basins were supported by a power >95% of most loci to reject panmixia. Genotypic disequilibrium could be taken as a consequence of selection [62], but only 4 out of 25 microsatellites with a deficit of heterozygotes showed signatures of directional selection. Therefore, selection cannot be endorsed as the cause of the generalized HW disequilibrium observed across loci and samples. Its most parsimonious explanation is a strong Wahlund effect [63] prompted by the spatiotemporal population dynamics of hake as an HMMS. For instance, this species is characterized by high reproductive variance and cohort overlapping [64], as well as by sporadic population admixtures of demes [65]. It is noteworthy that none of the well-known triggers of HW disequilibrium (gene flow, germinal mutation, selection, drift, and panmixia) can be detected with di-allelic SNPs ascertained to fit the HWE [24]. Curiously, if polymorphisms within EST loci ascertained for HWE were indeed adaptive, they cannot be used to infer selection because they are at post-selection genotypic equilibrium.

Under the hypothesis of basin-associated selection, we would expect a match of directional loci to sequence entries in genomic databases. However, the single-directional microsatellite between basins did not match any known protein, and none of the entries matching proteins (14%) showed signatures of selection. Those results agree with previous studies in hake, where most *F*_ST_ outliers from EST SNPs were found within basins, belonged to distinct genes between basins, consisted of synonymous polymorphisms and had no functional ascription to protein entries [24,29]. Those polymorphic properties make it unaffordable to test for differential allele functionality either between basins or among regions [66], and they led us to suspect that synonymous SNP changes exhibiting outlier *F*_ST_ can be explained by drift and allele surfing better than by directional selection [67]. Moreover, an indirect support given to the putative adaptive role of outlier SNPs comes from the significant regression reported between allele frequencies and both seawater salinity and surface temperature [21,24], while hake is a demersal species. Inferring adaptive scenarios in landscape genetics is risky [68] because allele frequencies fitting a latitudinal IBD range in the Atlantic hake metapopulation [57] are expected to correlate non-causally with many other latitudinal variables such as dawn time in winter and gravity [69].

### 4.2. Marker-Type-Dependent Structural Scenarios between Basins

While no differences of polymorphism were apparent attending to the cloning source of microsatellites, the significant differences among marker types (directional, balanced, neutral) showed three distinct structural scenarios of the same biological unit. The significant divergence observed between basins with 26 microsatellites (*F*_CT_ = 0.026) is congruent with previous figures using fewer microsatellites (0.029 [70]; 0.023 [57]; 0.023 [38]). These data suggest that the AOF is a likely phylogeographic barrier in hake [11,57] and occurs in many other taxa [71]. Indeed, neutral microsatellites have experienced widespread use in recent decades [13] because they provide information on the spatiotemporal distribution of genetic variation generated by the combined effect of mutation, drift, and gene flow. For instance, the distance afforded by 21 neutral markers provided a significant inter-basin divergence (*F*_CT_ = 0.028) (Table 2) that was very similar to that observed with the whole marker set (*F*_CT_ = 0.026). Noteworthy in this latter point is that the upwards outlier divergence of one directional marker (*F*_CT_ = 0.091) was canceled by the downwards outlier divergence of four balanced markers (*F*_CT_ = 0.005).

If divergence between basins is generated by drift in neutral loci or by selection and drift on directional loci and approaches zero in balanced loci, the current inter-basin scenario can be tested with several working hypotheses. First, under no effective barriers to gene flow except selection, directional loci will explain more variance between basins than neutral loci, with these latter offering a non-significant inter-basin divergence. Alternatively, under a barrier to gene flow and selection, both marker types would exhibit a significant divergence between basins, but that of directional loci would overwhelm the neutral one due to the synergic effect of selection and drift. This latter is the scenario observed after LOSITAN with directional loci exhibiting three-fold more inter-basin divergence (*F*_CT_ = 0.091) than neutral loci (*F*_CT_ = 0.024)**.** Likewise, two additional hypotheses can be tested by conjugating, balancing selection and drift. First, under no effective barriers to gene flow, neutral markers would exhibit a higher but non-significant variance between basins than balanced markers. Alternatively, under a barrier to gene flow, a significant variation between basins will come only from neutral markers. The latter scenario is the one observed with BAYESCAN, with neutral loci exhibiting a significant inter-basin divergence (*F*_CT_ = 0.031) but not so for balanced loci (*F*_CT_ = 0.005). Therefore, the structural scenario apprised will depend dramatically on the composition of the marker set; i.e., neutral markers and random admixtures of anonymous markers would average the genomic divergence between populations, balanced markers would deflate such divergence to near zero, and directional markers will inflate such divergence to a high gene-flow restriction [62].

Hake, as an HMMS, is under the strong influence of episodes of non-systematic drift provoked by a large reproductive variance between grounds, fishing mortality bottlenecks, and founder effects thereafter. Such drifting phenomena alternate with episodes of high spawning success and survival, intensive connectivity prompted by favorable marine currents, and overlapping generations, thus conforming to a large-scale metapopulation IBD scenario in the Atlantic [57]. The impact of such drift-triggers is expectedly high on low-polymorphic ESTs and protein-coding loci. Thereafter, the synergy between the AOF-mediated drift and the within-basin demographic drift on those low diversity regions is expected to produce directional selection-like outlier *F*_ST_-values [72]. Bayesian-inferred gene pools, which were systematically more conservative after BAPS [73] than after GENELAND, are a proof of such a drift-dependent scenario. For instance, because of inter-marker compensations, the global 26 microsatellite set portrayed the same number of gene pools (*k* = 2) as neutral markers and directional markers, while balanced markers portrayed a single gene pool. Therefore, an increasing number of gene pools is expected as we move from a balanced-skewed marker set (*k* = 1) towards a directional-skewed marker set (*k* = 2).

### 4.3. Within-Basin Substructuring and Management Implications

There are at least three contemporaneous visions of the European hake structure in the NE Atlantic. The first one is the two-stocks frame dealt with by ICES upon biomass and other management criteria. The second one is a complex scenario of non-delineated gene pools based on differences between French and Irish samples in protein-coding loci [26], between the Celtic Sea and Norwegian samples [70], or between Cantabrian and Portuguese waters, using microsatellites [70,74,75] and among the North Sea, Northern Portugal, and the rest of Atlantic regions, with ascertained outlier SNPs (*k* = 3 [24]; *k* = 4 [21]). Such fine-scale Atlantic ultrastructures are at odds with the third view of a quasi-panmictic scenario in the whole Atlantic metapopulation. This latter is supported by the higher divergence of samples from the range edges (the North Sea and the Canary Islands) upon a latitudinal IBD model, as depicted with random microsatellites [22,57], with a wide genomic SNPs coverage using RADseq [23] and with EST-microsatellites (this study). A similar conflicting scenario exists for the Mediterranean hake. Previous data from allozymes and mtDNA [26,76], geochemistry [11,38], and microsatellites [38,57] support a highly connective single Mediterranean metapopulation. However, up to three putatively locally adapted gene pools, namely Western, Central, and Eastern Mediterranean, have been reported using ascertained outlier SNPs [24]. The absence of both temporal consistency tests on such SNP patterns and the ascription of distant geographic samples to the same cluster (e.g., see Figure 3C in [24] and Figure 4 in [21]) do not allow those pools to be delineated temporally, spatially or conceptually.

### 4.4. Management Implications

Massive sequencing technologies are providing myriads of SNPs used as genomic markers for multiple purposes. Such an advancement has created a great excitement for the possibility of observing evolution in real time by natural selection. Additionally, the capability of substructuring fisheries using new genetic signatures is far more appealing than previous boring metapopulation scenarios of quasi-panmixia, as depicted with neutral and quasi-neutral genetic polymorphisms. However, mutation-drift-based neutral structuralism is as much real as putative SNP-based selective scenarios, provided they are insights on a single biological reality. In practice, we must decide what fishery partition is relevant to management, which one to sustainability, and which one to other uses such as commercial traceability. For instance, a commercial traceability system between basins could be validated if and only if the observed ascertained SNPs divergence [29] could be proven temporally stable [77]. However, the designation of multiple stocks within basins is an unfortunate decision for both management and sustainability, upon which we know of the biology of this species [65]. Ultrastructural views based on putative adaptive variation have little management value if regional divergence is a byproduct of reproductive drift on low polymorphic regions. Thus, a conservative approach to fishery management units should be adopted when a parsimonious, energetically cheaper, and biogeographically congruent, neutral explanation for population divergence can be enforced.

The assignment of hake samples using multilocus probabilities on distinct marker types [38] has a synergic advantage over HWE-FST-ascertained SNPs, of its ecumenicism in depicting metapopulation dynamics phenomena that are crucial for fishery management. By fixing our attention into punctual divergence between samples from ascertained SNPs instead of into commonalities, we can divide fisheries into fake evolutionary units and management units, that is, with the lower genetic delineation limit being the unique individual multilocus genotype. For instance, in hake, by breaking down the current 26-microsatellite set per marker type (see Table 2), one could claim the existence of multiple divergent stocks within the basin upon the 15% directional markers (*F*_SC_ = 0.037). However, using the 23% balanced loci among regions (*F*_SC_ = 0.012), one could claim a general lack of strong directional trends across the species range. Additionally, using the 62% neutral markers subset (*F*_SC_ = 0.020), one could claim that divergence among regions is significant but likely temporally evanescent under an IBD scenario in an HMMS metapopulation exhibiting a sweepstake-like reproductive model [78].

The poor agreement between outlier detection methods prevents endorsing *F*_ST_ outliers as an unequivocal proof of selection. The consistent AOF barrier-dependent drift between Atlantic and Mediterranean populations plus the within-basin drifted IBD scenario are expected to be severe in low polymorphic systems [79]. Thereafter, the 3-fold higher divergence between basins exhibited by putative directional microsatellites can be the result of demographic drift events on post-selected allelic systems, which mimic ongoing selection pressure on adaptive polymorphisms.

Letting alone the difficulty of implementing several conservation plans (i.e., up to *k* = 7 gene pools reported so far) in the European hake range, claims to conserve as much genetic diversity as possible for sustainability are plausible. Given that what has been the target of selection that predates the actual Holocene climate stability epoch might not be the focus of selection under the ongoing global change, restricting the definition of the species-relevant genetic diversity for adaptation to ascertained SNPs or to directional EST sequences is a genetic reductionism. The genetic variation with future adaptive relevance is unknown, but balanced markers are crucial candidates for the species welfare and survival [80]. Those supergenes are a rich source of adaptive variation and evolutionary novelty [81] and add up to responding to future environmental challenges. A moderate fishing pressure, an implementation of timely fishing bans at spawning grounds, and a pan-European responsible awareness of shared stocks are good practices for sustainability.

## 5. Conclusions

The *k* = 2 gene pools observed with directional and neutral markers in the range analyzed imply that European hake forms a metapopulation of two main-basin-located geographic stocks with restricted connectivity across the Almería–Oran oceanographic front as a persistent barrier to gene flow [55]. As an HMMS species, hake exhibits an IBD scenario within basin, i.e., latitudinal in the Atlantic and longitudinal in the Mediterranean [22]. This conclusion is at odds with within-basin ultrastructural views supported by a non-functional covariation of allele frequencies with temperature and salinity [e.g. 69].

The counterbalancing forces between directional markers (15% of loci, *k* = 2) and balanced markers (23% of loci, *k* = 1) within the whole marker set (*k* = 2), composed mainly by neutral markers, offer the same structural scenario as neutral markers alone (62%, *k* = 2). Such a neutral majority is robust to the inadvertent inclusion of directional markers and balanced markers in the current random marker set.

Each marker type offers a specific goal-oriented task in fisheries research. While no biological meaning has been shown for outlier directional markers in HMMS [30], such loci could be useful in commercial traceability, provided their polymorphisms were shown to be spatially delineated and temporally stable [77]. Such directional marker systems could also be useful in the reconstruction of evolutionary histories because of the scars of past selection on their polymorphisms (i.e., FAPS). Such putative directional markers could be assumed to be post-selective, not only because of their low polymorphism [e.g., 72].but also because any experimental proof exists on their qualitative variation being relevant for actual or future adaptation.

In order to describe fisheries’ structuring, it is important that the marker set accumulates a recent history of mutation and drift and reflects connective structures (i.e., NAPS). In this regard, directional makers are useless at best and misleading at worst for stock delineation and demographic management of fisheries due to their ascertainment bias [15]. A multilocus assignment using a random admixture of marker types (e.g., Figure 1) or a large genome coverage with thousands of SNPs [e.g. 23] have the synergic advantage over ascertained SNPs of their ecumenicism at depicting crucial population phenomena to delineate management units.

A conservation challenge is to characterize the amount and distribution of polymorphisms from balanced markers for their role as evolutionary engines for fisheries’ sustainability (i.e., APS). Another challenge is to determine if the reduced power to resolve genetic structures exhibited by highly mutating microsatellites (currently ascribed to homoplasy, e.g. [82]) is the result of a deflated *F*_ST_ outcome from stabilizing selection at linked loci and/or at EST-microsatellites.

## Figures and Tables

**Table 1:** Selection tests from BAYESCAN and LOSITAN on two hierarchical levels: between two subpopulations and among five regions (see Figure 1). Significant *q*-values after FDR correction are bolded and suggest balancing selection. The *q*-value is the statistical probability that an *F*_ST_ was significantly different from zero (see [54]). Significant *p*-values after the FDR correction (*p* ≥ 0.9987) are bolded and suggest directional selection, i.e., the probability that a marker violates the evolutionary neutral model (0.05 < neutral evolution < 0.95, before the FDR correction).

	BayeScan (Balancing Selection)				Lositan (Directional Selection)			
	Atlantic vs. Mediterranean		Five Regions		Atlantic vs. Mediterranean		Five Regions	
Molecular Marker	*F*_ST_	*q*-Value	*F*_ST_	*q*-Value	*F*_ST_	*p*-Value	*F*_ST_	*p*-Value
Anonymous microsatellites								
*Mmer*-hk29	0.0388	0.3783	0.0657	0.2341	0.0358	0.9652	0.0468	0.9905
*Mmer*-hk34b	**0.0080**	**0.0019**	**0.0212**	**0.0001**	0.0052	0.5267	0.0278	0.9332
*Mmer*-hk3b	0.0427	0.2404	0.0666	0.3780	**0.0883**	**0.9992**	**0.0702**	**0.9990**
*Mmer*-hk9b	**0.0057**	**0.0000**	**0.0134**	**0.0000**	0.0035	0.5174	0.0135	0.6675
*Mmer*-hk20	**0.0118**	**0.0232**	**0.0154**	**0.0000**	0.0079	0.6143	0.0088	0.1896
EST-microsatellites								
*Mmer*-EST_1.1	0.0193	0.7856	0.0236	0.2094	0.0716	0.9976	0.0448	0.9805
*Mmer*-EST_11.2	0.0106	0.1563	**0.0123**	**0.0154**	0.0149	0.8418	0.0159	0.6808
*Mmer*-EST_11.4	0.0202	0.6186	0.0278	0.5875	0.0330	0.9322	0.0189	0.5675
*Mmer*-EST_10.1	0.0191	0.7434	0.0277	0.4736	0.0267	0.7689	0.0379	0.8114
*Mmer*-EST_13.1	0.0185	0.6490	0.0276	0.5076	0.0267	0.8155	0.0227	0.6193
*Mmer*-EST_10.2	0.0188	0.7767	0.0233	0.2656	0.0128	0.6488	0.0122	0.3886
*Mmer*-EST_13.3	0.0183	0.5818	**0.0085**	**0.0228**	0.0136	0.6440	0.0023	0.0793
*Mmer*-EST_9.1	0.0258	0.4060	0.0748	0.1050	0.1307	0.9972	**0.1190**	**0.9998**
*Mmer*-EST_14.2	0.0221	0.4754	0.0551	0.0834	0.0442	0.9832	**0.0519**	**0.9987**
*Mmer*-EST_1.3	0.0187	0.7667	0.0279	0.5642	0.0166	0.7701	0.0268	0.8609
*Mmer*-EST_14.4	0.0184	0.6743	0.0271	0.3316	0.0175	0.7887	0.0339	0.9178
*Mmer*-EST_8.1	0.0188	0.7293	0.0282	0.3874	0.0126	0.6657	0.0318	0.8890
*Mmer*-EST_8.2	0.0197	0.6955	0.0282	0.4341	0.0203	0.7983	0.0382	0.9459
*Mmer*-EST_8.3	0.0197	0.7136	0.0282	0.6083	0.0295	0.9056	0.0306	0.9053
*Mmer*-EST_8.4	0.0142	0.3072	0.0153	0.0542	0.0085	0.5983	0.0167	0.6904
*Mmer*-EST_6.3 ^†^	**0.0046**	**0.0033**	**0.0075**	**0.0001**	0.0020	0.3831	0.0092	0.2386
*Mmer*-EST_6.4	0.0187	0.8017	0.0279	0.6601	0.0447	0.9853	**0.0537**	**0.9997**
*Mmer*-EST_9.4	0.0192	0.7939	0.0277	0.5378	0.0233	0.9137	0.0214	0.8320
*Mmer*-EST_2.2	0.0181	0.5358	0.0280	0.6445	0.0092	0.6133	0.0330	0.9786
*Mmer*-EST_4.3	0.0110	0.2150	0.0188	0.1373	0.0070	0.5505	0.0144	0.5493
*Mmer*-EST_2.4	0.0186	0.7557	0.0280	0.6273	0.0090	0.5873	0.0251	0.7479

^†^ This locus *F*_ST_ is significant but lacks falsifying power between subpopulations (see Table S3).

**Table 2:** Global molecular variation of microsatellite polymorphisms between the Atlantic and the Mediterranean hake populations, i.e., basins (*F*_CT_ = 0.026 *) and among five regions (*F*_SC_ = 0.020 *) as broken down per loci of subsets classified per selective type (directional, balanced, neutral). * Significant divergence at *p* < 0.01; ^ns^, non-significant *p*-value. Footnote symbols ^¶^ and ^»^ indicate putative aberrant *k*-pool scenarios regarding previous demographic and genetic data in this species.

	Directional Loci (LOSITAN)	Neutral Loci (LOSITAN)	Balanced Loci (BAYESCAN)	Neutral Loci (BAYESCAN)	Neutral Loci (Both Algorithms)
Between basins					
No. loci = 26 (100%)	1 (4)	25 (96)	4 (16)	22 (84)	21 (80)
*F*_CT_ = 0.026 *	0.091 *	0.024 *	0.005 ^ns^	0.031 *	0.028 *
*F*_CT_ change (%) Causal loci	+250 1 directional	−8 4 balanced	−81 4 balanced	+19% 1 directional	+ 8% neutral
BAPs pools (k) ^†^	2	1	1	1	1
Geneland pools (*k*) ^†^	2	2	1	2	2
Among regions ^‡^					
No. loci = 26 (100%)	4 (15%)	22 (85%)	6 (23%)	20 (77%)	16 (62%)
*F*_SC_ = 0.020 *	0.037 *	0.018 *	0.012 *	0.023 *	0.020 *
*F*_CT_ change (%) Causal loci	+ 83% 4 directional	−10 6 balanced	−40% 6 balanced	+15 4 directional	0 neutral
BAPs pools (k) ^§^	2 ^¶^	1	1	1	1
Geneland pools (*k*) ^§^	3 ^¥^	1	2 ^»^	2	2

^†^ Gene pool *k* = 1 indicates a single hake genetic unit along the Atlantic and the Mediterranean, *k* = 2 indicates a significant genetic split between those basins. ^‡^ Regional sample codes (Figure 1): NAN, North Atlantic North; CAN, Central Atlantic North; SAN, South Atlantic North; MEW, Western Mediterranean Sea; MEC, Central Mediterranean Sea. ^§^ Gene pool *k* = 1 indicates a single hake genetic unit, *k* = 2 indicates a significant split between Atlantic regions (NAN, CAN, SAN) and Mediterranean regions (MEW, MEC). ^¶^ Gene pool *k* = 2 indicates a significant split between Atlantic regions (NAN, CAN) and the Mediterranean regions (MEW, MEC) but including the Atlantic Gulf of the Cadiz region (SAN) in the Mediterranean (MEW, MEC). ^¥^ Gene pool *k* = 3 indicates a significant split between 3 groups of regions: Atlantic North (NAN, CAN), Gulf of Cadiz (SAN), and Mediterranean Sea (MEW, MEC). ^»^ Gene pool *k* = 2 indicates a significant split between the Central Mediterranean (MEC) and the rest of regions (NAN, CAN, SAN, MEW).

## Data Availability

Raw sequence reads were accessed from http://www.ebi.ac.uk/ena/data/view/ERP000950 on 22 May 2020.

## Supplementary Materials

The following supporting information can be downloaded at: www.mdpi.com/article/10.3390/ani12111462/s1, Figure S1: Roadmap for EST-microsatellite annotation from a transcriptomic cDNA library of the European hake and design of PCR multiplexes (see Table S1); Table S1: Primer sequences and gene diversity of microsatellite markers from *Merluccius merluccius*: *Mmer*-hk, anonymous markers; *Mmer*-EST, 23 new EST-microsatellites characterized in this study; Table S2: Sequence similarity search for 26 microsatellite-containing sequences (5 random clones and 23 EST-contigs) of *Merluccius merluccius*. Accession codes are given in the third column as GenBank for anonymous microsatellites and the European Nucleotide Archive for EST-microsatellites. Microsatellite-containing sequences were compared against fish protein sequence databases available from the National Center for Biotechnology Information (NCBI, 22 May 2020). Accession No. of matched coding sequences are given in the tenth column. NPM, no protein match was found. Table S3: Statistical power (Pearson, chi-square method of POWSIM) of marker subsets (see Table 1) according to their cloning source (R, randomly cloned microsatellites; EST, transcriptome-diverted microsatellites) as well as to their selective classification after LOSITAN (directional selection) and BAYESCAN (balancing selection). Table S4: Source of microsatellites (R, anonymous or random loci; EST, EST-loci) and their selective classification after two *F*_ST_-outlier based algorithms, i.e. LOSITAN (neutral polymorphism *vs.* selectively diversified polymorphism); BAYESCAN (neutral polymorphism *vs*. selectively balanced polymorphism). Superscripts d to g indicate putative aberrant *k*-pool scenarios regarding previous demographic and genetic data in this species. Table S5: Hierarchical AMOVA on Atlantic and Mediterranean hake regions and basins using nine subsets of microsatellites as classified per source (random loci and EST-diverted) as well as per selective role after LOSITAN (neutral loci *vs*. directional loci); SS, sum of squares; VC, variance component; %, percentage of variation; *F*, fixation index. * indicates that the probability of the observed value was equal or smaller than expected by random (*p* ≤ 0.01); ns, non-significant *p*-value. Table S6: Hierarchical AMOVA on Atlantic and Mediterranean hake regions and basins using nine subsets of microsatellites as classified per origin (random loci or EST-loci) as well as per selective role after BAYESCAN (neutral polymorphism *vs*. balanced polymorphism). SS, sum of squares; VC, variance component; %, percentage of variation; *F*, fixation index. * indicates that the probabilities of the observed values were equal or smaller than expected by random (*p* ≤ 0.01); ns, non-significant *p*-value.
