# Supplementary material for: EST-Microsatellite Types and Structural Scenarios in European Hake Fisheries
Source: Animals (Basel). 2022 Jun 4;12(11):1462. doi: 10.3390/ani12111462 (PMC9179439; doi:10.3390/ani12111462)
Supplement: Supplementary file 1 [file animals-12-01462-s001.zip › animals-1737691-supplementary.pdf]

Article

EST-Microsatellite Types and Structural Scenarios in European Hake Fisheries

Alfonso Pita †, María Fernández-Míguez † and Pablo Presa *

Laboratory of Marine Genetic Resources (ReXenMar), Centro de Investigación Mariña, Universidade de Vigo, Vigo 36310, Spain; alpita@uvigo.es (A.P.); mariafernandezm@uvigo.es (M.F.-M.)

* Correspondence: pressa@uvigo.es (P.P.)

† These authors contributed equally to this work.

Table S1. Primer sequences and gene diversity of microsatellite markers from *Merluccius merluccius*: *Mmer*-hk, anonymous markers; *Mmer*-EST, 23 new EST-microsatellites characterized in this study.

PCR	Locus	Primer sequences (5'- 3')	Repeat motif	T (°C) – $MgCl_2$ [mM]	Population	N	N_a	H_O	H_E	F_{IS}	Allele size range (bp)
**Anonymous microsatellites**											
Simplex	*Mmer*-hk29	F: 6-FAM GGGTTGCCAGTGGTTACGGGTGTG	$(GT)_{12}$	55 - 1.0	Atlantic Ocean	81	15	0.370	0.901	0.589*	138-172
		R: CGGCGACTGCGTGTTTCTGCTGCTGTG			Mediterranean Sea	62	13	0.403	0.827	0.512*	
Simplex	*Mmer*-hk34b	F: NED TATTGTCWATCTCTCTATCGGTGA	$(GT)_{24}$	55 - 1.7	Atlantic Ocean	107	22	0.654	0.917	0.287*	106-152
		R: AAAAAGATGCGAGGGACAGCTAAA			Mediterranean Sea	78	19	0.526	0.921	0.429*	
Triplex 1	*Mmer*-hk3b	F: 6-FAM ACCCGGTCTCCTCACTAGCAGTTT	$(GT)_{17}$	55 - 1.5	Atlantic Ocean	85	11	0.824	0.839	0.018	322-348
		R: AGGCTTCAGGAACAGAATGCTATG			Mediterranean Sea	75	10	0.600	0.682	0.120*	
	Mmer-hk9b	F: HEX CAAGCAAGACCTGGAGGGGAAAGAGATG	$(GA)_{23}$		Atlantic Ocean	95	29	0.642	0.960	0.331*	121-181
		R: GTGCGTGCCTCCCAGGAGTTGCTTGTAG			Mediterranean Sea	79	29	0.766	0.958	0.200*	
	Mmer-hk20	F: NED TCGGCTGCAGGTCAGTTCAGTTTTT	$(GT)_{13}$		Atlantic Ocean	108	19	0.806	0.911	0.116*	203-255
		R: GTGCCTGTTTTCCTGCCATGCT			Mediterranean Sea	81	17	0.815	0.882	0.076*	
**EST-microsatellites**											
Triplex 2	*Mmer*-EST_1.1	F: 6-FAM CGTGTCGTAAACTCGCGTC	$(AAG)_{10}$	60 - 1.5	Atlantic Ocean	93	12	0.688	0.763	0.099	213-255
		R: ACACAGCGTTGCATAAAGCG			Mediterranean Sea	47	17	0.681	0.913	0.255*	
	Mmer-EST_11.2	F: HEX GGATACAGGGAGTAACCGTGG	$(AC)_8$		Atlantic Ocean	106	27	0.755	0.927	0.186*	140-210
		R: CGCCAATCTGCATTATCTGC			Mediterranean Sea	45	30	0.778	0.965	0.194*	
	Mmer-EST_11.4	F: 6-FAM AGCGGTTCTGGTTGAGAGGT	$(CCG)_6$		Atlantic Ocean	94	10	0.777	0.834	0.069*	91-127
		R: ACAAACGCCACCTTCCAGT			Mediterranean Sea	57	13	0.667	0.906	0.264*	
Tetraplex 1	*Mmer*-EST_10.1	F: 6-FAM GTCATCATCCTCATCCTC	$(AGT)_6$	58 - 1.6	Atlantic Ocean	97	10	0.423	0.590	0.284*	225-261
		R: CTACCTCCTCGTAGAAACAC			Mediterranean Sea	66	9	0.379	0.636	0.405*	
	Mmer-EST_13.1	F: HEX GCCCGCACAGTCACAGGT	$(AC)_{10}$		Atlantic Ocean	101	10	0.713	0.776	0.082	190-210

		R: CAAATCCCATGACAAAGCCA			Mediterranean Sea	73	10	0.534	0.660	0.191*	
	Mmer-EST_10.2	F: $^{6\text{-}FAM}$TACCCAAATGCCAGCAAATC	(AC)$_8$		Atlantic Ocean	92	10	0.717	0.803	0.106	177-201
		R: TTTAGGGATTCACACGTCAGC			Mediterranean Sea	68	11	0.677	0.765	0.116	
	Mmer-EST_13.3	F: ^{HEX}TGAAAGCGTTGGTGTTGAAG	(AAC)$_6$		Atlantic Ocean	97	9	0.804	0.826	0.026	126-159
		R: CATTTGTTCATTCCCAGCTCA			Mediterranean Sea	70	11	0.643	0.804	0.200*	
Tetraplex 2	*Mmer*-EST_9.1	F: $^{6\text{-}FAM}$CTGGTCCCAAATAAATCCT	(AC)$_6$	55 - 1.8	Atlantic Ocean	83	10	0.301	0.584	0.484*	216-246
		R: TTGAATGGTGCTACTTGATT			Mediterranean Sea	68	6	0.324	0.610	0.470*	
	Mmer-EST_14.2	F: ^{HEX}ATTCCCACTGTTCGGCTGT	(AC)$_{10}$		Atlantic Ocean	84	18	0.571	0.891	0.358*	162-184
		R: GTTTGACTCGGAAACCATGC			Mediterranean Sea	56	12	0.500	0.881	0.432*	
	Mmer-EST_1.3	F: $^{6\text{-}FAM}$TCTCCGTGGTAGATGGCTCA	(AAT)$_{14}$		Atlantic Ocean	45	17	0.667	0.901	0.260*	114-150
		R: AAATTGCTGCCGACAAATAAGT			Mediterranean Sea	37	17	0.703	0.893	0.213*	
	Mmer-EST_14.4	F: ^{HEX}AGCAAGGAATGTAAACCGCA	(AG)$_7$		Atlantic Ocean	82	28	0.598	0.903	0.338*	78-122
		R: GCGGGAGACAAGAGGTGTT			Mediterranean Sea	64	18	0.594	0.803	0.261*	
Tetraplex 3	*Mmer*-EST_8.1	F: $^{6\text{-}FAM}$CGTTGGTACACTTTGCTACTC	(AC)$_{14}$	60 - 1.5	Atlantic Ocean	36	15	0.806	0.887	0.092	198-226
		R: GAGCCCTTCTCTTCCTCTG			Mediterranean Sea	25	9	0.640	0.823	0.222*	
	Mmer-EST_8.2	F: ^{HEX}CAGCAACAAACGCAAACTGA	(AC)$_9$		Atlantic Ocean	40	11	0.600	0.859	0.302*	150-174
		R: GATGGTCGTTGGATCGTCTC			Mediterranean Sea	25	7	0.760	0.798	0.047	
	Mmer-EST_8.3	F: $^{6\text{-}FAM}$CTCCTCCACTACCCACCTGA	(ACG)$_6$		Atlantic Ocean	91	18	0.670	0.897	0.252*	108-132
		R: AACCATCATCTGCCACCATC			Mediterranean Sea	79	13	0.608	0.842	0.278*	
	Mmer-EST_8.4	F: ^{HEX}CCATTCGGTAAGTCAGTCGG	(AAGT)$_7$		Atlantic Ocean	98	34	0.796	0.952	0.164*	60-110
		R: TGAACATCGGTTAGGTCCACA			Mediterranean Sea	68	22	0.750	0.915	0.180*	
Tetraplex 4	*Mmer*-EST_3.1	F: $^{6\text{-}FAM}$CGCTATGCGTTATGGGAGAA	(AT)$_6$	60 - 1.5	Atlantic Ocean	28	9	0.536	0.827	0.353*	192-212
		R: CCCAGAGGTTCAAATCCAAA			Mediterranean Sea	0	0	NA	NA	NA	
	Mmer-EST_6.3	F: ^{HEX}TGTTGCCGCTGTCTAGCTTA	(AGG)$_7$		Atlantic Ocean	110	33	0.909	0.935	0.028*	122-170

		R: AGCATCAAAGAAGAAACCGTG			Mediterranean Sea	79	27	0.772	0.922	0.162*	
	Mmer-EST_6.4	F: $^{\text{6-FAM}}$CTTTCCCTTTAGTTTGCCCG	(CCG)$_6$		Atlantic Ocean	82	30	0.707	0.936	0.244*	87-122
		R: GACGACACGTTGTTCCGAC			Mediterranean Sea	72	24	0.736	0.835	0.119*	
	Mmer-EST_9.4	F: ^{HEX}TTGTAGCTGTATCTGCTTTAATGA	(ACAT)$_{11}$		Atlantic Ocean	57	29	0.597	0.941	0.366*	82-122
		R: TGTTCAATGTGTTTCTGTAATCC			Mediterranean Sea	30	13	0.300	0.903	0.668*	
Tetraplex 5	*Mmer*-EST_2.1	F: $^{\text{6-FAM}}$GACGGCAACCACAACGATA	(CCG)$_8$	59 - 1.5	Atlantic Ocean	57	29	0.474	0.950	0.501*	188-234
		R: TGCACAACCACACCACAATA			Mediterranean Sea	19	10	0.474	0.882	0.463*	
	Mmer-EST_2.2	F: ^{HEX}CCCGAAACAACATTTCCAGA	(AAT)$_6$		Atlantic Ocean	92	28	0.587	0.913	0.357*	140-185
		R: GTTTGGCTCAGAAGACCCTG			Mediterranean Sea	71	18	0.352	0.929	0.621*	
	Mmer-EST_4.3	F: $^{\text{6-FAM}}$GCGTCTCAGGATAAGTTGGG	(AAC)$_{10}$		Atlantic Ocean	102	28	0.726	0.939	0.228*	115-149
		R: TTGACATTCAGGACAAGCAGA			Mediterranean Sea	79	22	0.722	0.932	0.226*	
	Mmer-EST_2.4	F: ^{HEX}CGATTCACGAAGAGCAGTCA	(AAC)$_6$		Atlantic Ocean	86	22	0.733	0.846	0.134*	76-108
		R: ACGACCTTTAGCCGGTCTGT			Mediterranean Sea	78	19	0.654	0.867	0.246*	

NOTE. F, forward sequence; R, reverse sequence; T (°C), annealing temperature; $MgCl_2$ [mM], $MgCl_2$ concentration; N, number of individuals genotyped per locus and population; N_a, number of alleles per locus; H_O, observed heterozygosity; H_E, expected heterozygosity; F_{IS}, intrapopulation fixation index [82]; NA, no amplification observed; * p ($F_{IS} = 0$) ≤ 0.01.

Table S2. Sequence similarity search for 26 microsatellite-containing sequences (5 random clones and 23 EST contigs) of *Merluccius merluccius*. Accession codes are given in the third column as GenBank for anonymous microsatellites and the European Nucleotide Archive for EST microsatellites. Microsatellite-containing sequences were compared against fish protein sequence databases available from the National Center for Biotechnology Information (NCBI, 22 May 2020). Accession No. of matched coding sequences are given in the tenth column. NPM, no protein match was found.

Multiplex	Locus	GenBank accession No.	Description	Max score	Total score	Query cover	E-value	Identity	Accession No. of matched sequences	Clone sequence (microsatellite tracks are bolded)
**Anonymous microsatellites**										
Simplex	Mmer-HK29	AF137597	Uncharacterized protein LOC100002239 precursor [*Danio rerio*]	27.7	27.7	45%	6.1	32%	NP_001091729.1	GGGTTGCCAGTGGTTACGG**GTGTGTGTGTGTGTGTGT GTGTGT**GGGGTGGGGGTGGGGGTGGGGGGGGGGGGT CTCTTTTGGGCTGATAATACTATACAGTACAGGAGGGA AAAACACACAAGGTCTAGCTGGATACACAGCAGCAGAA ACACGCAGTCGCCG
Simplex	Mmer-HK34b	AF137595	NPM							TATTGTCWATCTCTCTATCGGTGACCGGCGTGGGGGGA GGGGAGGGGGGGG**GTGTGTGTGTGTGTGTGTGTGTGTG TGTGTGTGTGTGTGTGTGTGTGT**GGGGGGGGAAA TCACTTTAGCTGTCCCTCGCATCTTTTT
Triplex 1	Mmer-HK3b	AF136627	NPM							ACCCGGTCTCCTCACTAGCAGTTTGTGTATGGAGCCAGG GGTGTGTTCGGATTACGCAGATTATTCAATCAATTCATA GTAACGCACCGCATTTAACGTTCAGTAATGATAACAGC GTGGTAACGACGGAACAGTAATTAGTTAGATTACCCGC TACTGGAAAATAACGGCGTTACATAACGCATTCTTTTAA ACGGCGTTATTCCAAACACTGCAAGGGGACACATCTGG GTTT**GTGTGTGTGTGTGTGTGTGTGTGTGTGTGTGTG T**CCCTCTCACCTTTGGATTGACTATAATCTCCATGTCAT AGCATTCTGTTCCTGAAGCCT
	Mmer-HK9	AF136628	NPM							CAAGCAAGACCTGGAGGGGAAAGAGAT**GAGAGAGAG AGAGAGAGAGAGAGAGAGAGAGAGAGAGAGAGAGAGA GA**TAGTGATGGGCAGGTAAGCACAATGGGCTACAAGCA ACTCCTGGGAGGCACGCAC
	Mmer-HK20	AF137596	cilia- and flagella-associated protein 44 [*Gadus morhua*]	35.4	67.4	38%	0.009	81%	XP_030204510.1	TCGGCTGCAGGTCAGTTCAGTTTTTGGCCAAAWAGTCA AAAACCTTGACAGAGCCCTGCAAAGGAGACAGAAGTAT ATGTACATCATTCT**GTGTGTGTGTGTGTGTGTGTGTG TGT**CATAGAGTTACTACACTCACAGTCCAGTGCGGTAG TGGCCATCAGGTGGCTTGCACGGAAACGTCCATTGCCT GGATGGCCCCAGCATGGCAGGAAAACAGGCAC

LOW QUALITY PROTEIN: cilia- and flagella-associated protein 44 [*Electrophorus electricus*]	32.3	60.8	38%	0.53	81%	XP_026863865.1
LOW QUALITY PROTEIN: cilia- and flagella-associated protein 44 [*Scleropages formosus*]	32.0	60.1	35%	1.2	87%	XP_029104418.1
cilia- and flagella-associated protein 44 [*Clupea harengus*]	33.5	59.3	36%	2.0	88%	XP_031440333.1
Hypothetical protein AMELA_G00222490 [*Ameiurus melas*]	32.3	58.9	36%	2.9	81%	KAF4075757.1
cilia- and flagella-associated protein 44 [*Sphaeramia orbicularis*]	32.0	58.1	37%	4.9	75%	XP_030005630.1
cilia- and flagella-associated protein 44 [*Parambassis ranga*]	32.7	57.8	37%	6.0	68%	XP_028274026.1
cilia- and flagella-associated protein 44 [*Gadus morhua*]	35.4	67.4	38%	0.009	81%	XP_030204510.1
Cilia- and flagella-associated protein 44 [*Seriola dumerili*]	32.3	57.4	37%	5.1	68%	XP_022600157.1
Cilia- and flagella-associated protein 44 [*Seriola lalandi dorsalis*]	32.3	57.4	37%	5.1	68%	XP_023281359.1
Cilia- and flagella-associated protein 44 [*Monopterus albus*]	32.3	57.4	37%	5.1	68%	XP_020456723.1
cilia- and flagella-associated protein 44 isoform X1 [*Oreochromis aureus*]	30.4	57.4	38%	8.3	63%	XP_031601520.1
cilia- and flagella-associated protein 44 isoform X2 [*Oreochromis aureus*]	30.4	57.4	38%	8.3	63%	XP_031601522.1
cilia- and flagella-associated protein 44 isoform X1 [*Pseudochaenichthys georgianus*]	32.3	57.0	37%	7.0	81%	XP_033952235.1
cilia- and flagella-associated protein 44 isoform X1 [*Trematomus bernacchii*]	32.3	57.0	37%	7.0	81%	XP_033981538.1

			cilia- and flagella-associated protein 44 isoform X2 [*Pseudochaenichthys georgianus*]	32.3	57.0	37%	7.0	81%	XP_033952236.1	
			cilia- and flagella-associated protein 44 isoform X2 [*Trematomus bernacchii*]	32.3	57.0	37%	7.0	81%	XP_033981539.1	
			LOW QUALITY PROTEIN: cilia- and flagella-associated protein 44 [*Gymnodraco acuticeps*]	32.3	57.0	37%	7.0	81%	XP_034066690.1	
			PREDICTED: cilia- and flagella-associated protein 44 [*Notothenia coriiceps*]	32.3	56.6	37%	7.9	81%	XP_010790959.1	
			Cilia- and flagella-associated protein 44 [*Danio rerio*]	29.3	29.3	17%	3.0	85%	XP_017209920.2	
**EST-microsatellites**										
Triplex 2	Mmer-EST_1.1	ERR048980.48818	NPM							GAGGCGCCGTGTCGTAAACTCGCGTCACCCGTACAGCC<br>GCCAACGCCGCCTGAGGAGCCGCCAACGCCGCCTGAGG<br>AGACGCCATCACTCAGCTTTCCGCTCCTCACGATGCAGT<br>AGCCAATCAGGCGATAGGACCGCACCGCTGTAGCCAAT<br>CATGT**TTCTTCTTCTTCTTCTTCTTCTTCTTCTTC**TGG<br>TCGGGCGCCGCGGGCAGACCTCCCGGCGCCGCTTTATG<br>CAACGCTGTGTTG
	Mmer-EST_11.2	ERR048979.15436	NPM							GTCATGTACACTACAGGATACAGGGAGTAACCGTGGTT<br>ACTCAGAC**TGTGTGTGTGTGTGTG**TTTTACACGTCTGG<br>CCGTCTTGGCTGACCTGGTGGACGAGGGGTTAACTGCTC<br>ACTAGTGATGTATTTATTAAAGAACACGTAATGCAGAT<br>AATGCAGATAATGCAGATAATGCAGATTGGCGTTTGGC<br>TGCGGTGCGGATGTATTCGGTGGAGTGGAGACG
	Mmer-EST_11.4	ERR048976.13483	PR domain zinc finger protein 2-like [*Gadus morhua*]	43.9	43.9	94%	0.006	44%	XP_030211173.1	TAGGCCGAGGTTCTCCTCCGAGATGTTGCTGGAGACGTC<br>CAGCATGTAGTGCTGCTCGGCGCGTTCTGCAGGACCGTC<br>TGCAGGAGCCGCACAGCCAGGCAGCGGTTCTGGTTGAG<br>AGGTCATGCCGGCCGCT**CCGCCGCCGCCGCCGCCG**CT<br>CGCCTGGAGCGGTGGCGTCCGGTCGGCCTGCTCGACTG<br>GAAGGTGGCGTTTGTGAACCCGGGCGCTCGTGCCG
			PR domain zinc finger protein 2-like [*Scleropages formosus*]	37.7	37.7	94%	1.1	37%	XP_018592109.2	

			PR domain zinc finger protein 2-like [*Scleropages formosus*]	37.7	37.7	94%	1.1	37%	KPP72719.1	
			PR domain zinc finger protein 2-like [*Anabas testudineus*]	35.8	35.8	94%	4.7	45%	XP_026224024.1	
			PR domain zinc finger protein 2 isoform X2 [*Larimichthys crocea*]	35.0	35.0	94%	7.7	41%	XP_019115632.2	
			PR domain zinc finger protein 2 isoform X1 [*Larimichthys crocea*]	35.0	35.0	94%	7.7	41%	XP_010730713.3	
Tetraplex 1	Mmer-EST_10.1	ERR048980.25012	transmembrane emp24 domain-containing protein 3-like [*Oncorhynchus nerka*]	50.8	50.8	29%	3.00E-07	92%	XP_029507984.1	GTCATCATCCTCATCCTC**AGTAGTAGTAGTAGTAGTAGT**ACTATCATCTGGGTCTGTACCCCATCCTCCTCATCCTCACTGCCGTGTGGTCCGGTCCCCTCCTGACCATGCGTAGCCTGGGGTTTAGCTGCGTCCCGGTCCCGGTCCTGGTCCTGGTCCTGCATGTGGTCCTGGTGGGGAGCACGGAGCTCACGTTTGAGCTGCCCGACAATGATAAGCAGTGTTTCTACGAGGAGGTAGAAAACGGTGTG
			transmembrane emp24 domain-containing protein 3-like [*Salmo trutta*]	51.6	51.6	29%	4.00E-07	92%	XP_029600861.1	
			transmembrane emp24 domain-containing protein 3-like [*Oncorhynchus tshawytscha*]	51.6	51.6	29%	4.00E-07	92%	XP_024264943.1	
			transmembrane emp24 domain-containing protein 3-like [*Gadus morhua*]	51.2	51.2	29%	5.00E-07	88%	XP_030232043.1	
			transmembrane emp24 domain-containing protein 3-like [*Myripristis murdjan*]	50.8	50.8	29%	9.00E-07	88%	XP_029935681.1	
			transmembrane emp24 domain-containing protein 3-like [*Sphaeramia orbicularis*]	48.9	48.9	27%	4.00E-06	91%	XP_029983726.1	
			transmembrane emp24 domain-containing protein 3-like [*Denticeps clupeoides*]	47.0	47.0	27%	2.00E-05	87%	XP_028824730.1	
			transmembrane emp24 domain-containing protein 3-like [*Boleophthalmus pectinirostris*]	47.0	47.0	27%	2.00E-05	87%	XP_020797312.1	
			transmembrane emp24 domain-containing protein 3-like [*Etheostoma spectabile*]	46.6	46.6	27%	3.00E-05	87%	XP_032374488.1	
			transmembrane emp24 domain-containing protein 3-like [*Perca flavescens*]	46.2	46.2	27%	3.00E-05	87%	XP_028437200.1	

transmembrane emp24 domain-containing protein 3-like [*Sander lucioperca*]	46.2	46.2	27%	4.00E-05	87%	XP_031169891.1
transmembrane emp24 domain-containing protein 3-like [*Carassius auratus*]	46.2	46.2	29%	4.00E-05	79%	XP_026123094.1
transmembrane emp24 domain-containing protein 3-like [*Monopterus albus*]	46.2	46.2	27%	4.00E-05	87%	XP_020475580.1
transmembrane emp24 domain-containing protein 3-like [*Trematomus bernacchii*]	46.2	46.2	27%	4.00E-05	87%	XP_033972434.1
transmembrane emp24 domain-containing protein 3-like [*Electrophorus electricus*]	46.2	46.2	30%	3.00E-05	76%	XP_026853677.1
transmembrane emp24 domain-containing protein 3-like [*Chanos chanos*]	45.8	45.8	27%	6.00E-05	83%	XP_030622302.1
transmembrane emp24 domain-containing protein 3-like [*Parambassis ranga*]	45.4	45.4	30%	6.00E-05	76%	XP_028258374.1
transmembrane emp24 domain-containing protein 3-like [*Periophthalmus magnuspinnatus*]	45.1	45.1	27%	1.00E-04	83%	XP_033846938.1
transmembrane emp24 domain-containing protein 3-like isoform X1 [*Seriola dumerili*]	45.1	45.1	27%	1.00E-04	83%	XP_022595865.1
transmembrane emp24 domain-containing protein 3-like [*Amphiprion ocellaris*]	45.1	45.1	27%	1.00E-04	83%	XP_023153366.1
transmembrane emp24 domain-containing protein 3-like [*Mastacembelus armatus*]	44.7	44.7	27%	1.00E-04	83%	XP_026149183.1
transmembrane emp24 domain-containing protein 3-like [*Archocentrus centrarchus*]	44.7	44.7	26%	2.00E-04	86%	XP_030580514.1
transmembrane emp24 domain-containing protein 3-like [*Clupea harengus*]	44.7	44.7	27%	2.00E-04	78%	XP_012697816.2
transmembrane emp24 domain-containing protein 3-like [*Tachysurus fulvidraco*]	44.7	44.7	27%	2.00E-04	78%	XP_027008813.1
transmembrane emp24 domain-containing protein 3-like [Echeneis naucrates]	44.3	44.3	27%	2.00E-04	83%	XP_029355001.1

transmembrane emp24 domain-containing protein 3-like [*Takifugu rubripes*]	44.3	44.3	27%	2.00E-04	74%	XP_003969553.1
transmembrane emp24 domain-containing protein 3-like [*Sparus aurata*]	43.9	43.9	27%	3.00E-04	78%	XP_030269428.1
transmembrane emp24 domain-containing protein 3-like [*Xiphophorus maculatus*]	43.9	43.9	27%	3.00E-04	83%	XP_005796202.1
transmembrane emp24 domain-containing protein 3-like [*Betta splendens*]	43.5	43.5	27%	4.00E-04	83%	XP_029000228.1
transmembrane emp24 domain-containing protein 3-like [*Paramormyrops kingsleyae*]	43.1	43.1	29%	5.00E-04	75%	XP_023697741.1
transmembrane emp24 domain-containing protein 3-like [*Seriola lalandi dorsalis*]	43.1	43.1	27%	5.00E-04	78%	XP_023267753.1
transmembrane emp24 domain-containing protein 3-like [*Scleropages formosus*]	42.7	42.7	27%	8.00E-04	74%	XP_018619294.2
transmembrane emp24 domain-containing protein 3-like [*Oryzias melastigma*]	42.7	42.7	29%	8.00E-04	75%	XP_024151815.1
transmembrane emp24 domain-containing protein 3 precursor [*Salmo salar*]	51.6	51.6	29%	4.00E-07	92%	NP_001139866.1
transmembrane emp24 domain-containing protein 3 precursor [*Oncorhynchus mykiss*]	51.6	51.6	29%	4.00E-07	92%	ACO08423.1
transmembrane emp24 domain-containing protein 3 precursor [*Esox lucius*]	50.8	50.8	29%	7.00E-07	92%	NP_001297835.1
transmembrane emp24 domain-containing protein 3 precursor [*Danio rerio*]	47.8	47.8	30%	1.00E-05	80%	NP_001122140.1
transmembrane emp24 domain-containing protein 3 precursor [*Osmerus mordax*]	47.4	47.4	29%	1.00E-05	88%	ACO09604.1
transmembrane emp24 domain-containing protein 3 [*Pangasianodon hypophthalmus*]	47.0	47.0	27%	2.00E-05	87%	XP_034164308.1
transmembrane emp24 domain-containing protein 3 [*Larimichthys crocea*]	46.2	46.2	27%	4.00E-05	87%	XP_010753799.1
transmembrane emp24 domain-containing protein 3 [*Epinephelus lanceolatus*]	45.8	45.8	27%	5.00E-05	87%	XP_033483227.1

transmembrane emp24 domain-containing protein 3 [*Astyanax mexicanus*]	45.8	45.8	27%	6.00E-05	83%	XP_022525710.1
transmembrane emp24 domain-containing protein 3 [*Fundulus heteroclitus*]	45.4	45.4	29%	6.00E-05	79%	XP_012724201.1
transmembrane emp24 domain-containing protein 3 [*Oreochromis niloticus*]	45.8	45.8	27%	6.00E-05	83%	XP_003437677.1
transmembrane emp24 domain-containing protein 3 [*Gymnodraco acuticeps*]	45.4	45.4	27%	7.00E-05	87%	XP_034052550.1
transmembrane emp24 domain-containing protein 3 [*Maylandia zebra*]	45.4	45.4	27%	8.00E-05	83%	XP_004539647.1
transmembrane emp24 domain-containing protein 3 [*Oryzias latipes*]	45.1	45.1	29%	9.00E-05	79%	XP_023809353.1
transmembrane emp24 domain-containing protein 3 isoform X2 [*Cottoperca gobio*]	44.3	44.3	27%	2.00E-04	83%	XP_029313050.1
transmembrane emp24 domain-containing protein 3 [*Pseudochaenichthys georgianus*]	44.3	44.3	27%	2.00E-04	78%	XP_033932429.1
transmembrane emp24 domain-containing protein 3 [*Bagarius yarrelli*]	43.1	43.1	27%	6.00E-04	74%	TSL68231.1
transmembrane emp24 domain-containing protein 3 [*Cyclopterus lumpus*]	40.0	40.0	27%	0.006	74%	XP_034414487.1
transmembrane emp24 domain-containing protein 3 [*Liparis tanakae*]	39.3	39.3	27%	0.011	74%	TNN50236.1
Isocitrate dehydrogenase (NADP), mitochondrial-like isoform X2 [*Trematomus bernacchii*]	48.5	48.5	27%	9.00E-06	87%	XP_033972435.1
Isocitrate dehydrogenase (NADP), mitochondrial-like isoform X2 [*Periophthalmus magnuspinnatus*]	47.4	47.4	27%	2.00E-05	83%	XP_033846944.1
Isocitrate dehydrogenase (NADP), mitochondrial-like [*Paramormyrops kingsleyae*]	47.0	47.0	27%	3.00E-05	78%	XP_023671162.1

Isocitrate dehydrogenase (NADP), mitochondrial-like isoform X2 [*Seriola dumerili*]	47.0	47.0	27%	3.00E-05	83%	XP_022595872.1
Isocitrate dehydrogenase (NADP), mitochondrial isoform X3 [*Cottoperca gobio*]	46.2	46.2	27%	5.00E-05	83%	XP_029313062.1
Isocitrate dehydrogenase (NADP), mitocondrial -like [*Acanthochromis polyacanthus*]	45.8	45.8	27%	7.00E-05	78%	XP_022055226.1
Isocitrate dehydrogenase (NADP), mitochondrial [*Channa argus*]	45.8	45.8	27%	7.00E-05	87%	KAF3686802.1
Isocitrate dehydrogenase (NADP), mitochondrial-like [*Kryptolebias marmoratus*]	44.7	44.7	27%	2.00E-04	74%	XP_017274110.2
Isocitrate dehydrogenase (NADP), mitochondrial-like [*Labrus bergylta*]	42.4	42.4	27%	0.001	78%	XP_020510597.1
Isocitrate dehydrogenase (NADP), mitochondrial-like [*Gouania willdenowi*]	40.4	40.4	27%	0.005	78%	XP_028329632.1
transmembrane emp24 domain-containing protein 7-like [*Electrophorus electricus*]	40.8	40.8	26%	0.004	73%	XP_026855530.1
transmembrane emp24 domain-containing protein 7-like [*Salarias fasciatus*]	40.4	40.4	27%	0.005	74%	XP_029961128.1
transmembrane emp24 domain-containing protein 7-like [*Carassius auratus*]	40.4	40.4	27%	0.005	70%	XP_026072871.1
transmembrane emp24 domain-containing protein 7-like [*Denticeps clupeoides*]	40.4	40.4	29%	0.005	71%	XP_028852684.1
transmembrane emp24 domain-containing protein 7-like [*Gouania willdenowi*]	40.4	40.4	26%	0.006	77%	XP_028312234.1
transmembrane emp24 domain-containing protein 7-like [*Betta splendens*]	40.0	40.0	27%	0.006	74%	XP_029017970.1
transmembrane emp24 domain-containing protein 7-like [*Xiphophorus maculatus*]	40.0	40.0	26%	0.007	73%	XP_005794935.1

transmembrane emp24 domain-containing protein 7-like [*Clupea harengus*]	39.7	39.7	27%	0.009	70%	XP_012691396.1
transmembrane emp24 domain-containing protein 7-like [*Xiphophorus couchianus*]	39.7	39.7	26%	0.009	73%	XP_027890072.1
transmembrane emp24 domain-containing protein 7-like [*Boleophthalmus pectinirostris*]	39.7	39.7	26%	0.009	73%	XP_020776251.1
transmembrane emp24 domain-containing protein 7-like [*Salmo trutta*]	39.7	39.7	26%	0.009	73%	XP_029617957.1
transmembrane emp24 domain-containing protein 7-like [*Oncorhynchus kisutch*]	39.7	39.7	26%	0.009	73%	XP_020345026.1
transmembrane emp24 domain-containing protein 7-like [*Oncorhynchus mykiss*]	39.7	39.7	26%	0.010	73%	XP_021460916.1
transmembrane emp24 domain-containing protein 7-like [*Oncorhynchus nerka*]	39.7	39.7	26%	0.010	73%	XP_029493005.1
transmembrane emp24 domain-containing protein 7-like [Echeines naucrates]	39.7	39.7	26%	0.010	73%	XP_029366955.1
transmembrane emp24 domain-containing protein 7-like isoform X2 [*Labrus bergylta*]	39.7	39.7	26%	0.011	73%	XP_020499123.1
transmembrane emp24 domain-containing protein 7-like isoform X1 [*Labrus bergylta*]	39.7	39.7	26%	0.011	73%	XP_020499122.1
transmembrane emp24 domain-containing protein 7-like isoform X2 [*Sparus aurata*]	39.3	39.3	26%	0.012	77%	XP_030272512.1
transmembrane emp24 domain-containing protein 7-like [*Monopterus albus*]	39.3	39.3	26%	0.012	73%	XP_020458067.1
transmembrane emp24 domain-containing protein 7-like isoform X2 [*Gadus morhua*]	39.3	39.3	26%	0.015	73%	XP_030214769.1
transmembrane emp24 domain-containing protein 7-like [*Scleropages formosus*]	39.3	39.3	26%	0.015	73%	KPP64488.1
transmembrane emp24 domain-containing protein 7-like [*Oryzias melastigma*]	38.9	38.9	26%	0.016	73%	XP_024131620.1

transmembrane emp24 domain-containing protein 7-like [*Kryptolebias marmoratus*]	38.9	38.9	26%	0.017	73%	XP_017269375.1
transmembrane emp24 domain-containing protein 7-like [*Cottoperca gobio*]	38.9	38.9	26%	0.017	73%	XP_029296333.1
transmembrane emp24 domain-containing protein 7-like [*Myripristis murdjan*]	38.9	38.9	26%	0.017	73%	XP_029916728.1
transmembrane emp24 domain-containing protein 7-like [*Perca flavescens*]	38.9	38.9	26%	0.017	73%	XP_028433080.1
transmembrane emp24 domain-containing protein 7-like [*Archocentrus centrarchus*]	38.9	38.9	26%	0.017	73%	XP_030593397.1
transmembrane emp24 domain-containing protein 7-like [*Parambassis ranga*]	38.9	38.9	26%	0.018	73%	XP_028270940.1
transmembrane emp24 domain-containing protein 7-like [*Mastacembelus armatus*]	38.9	38.9	26%	0.018	73%	XP_026178167.1
transmembrane emp24 domain-containing protein 7-like [*Anarrhichthys ocellatus*]	38.9	38.9	26%	0.018	73%	XP_031712030.1
transmembrane emp24 domain-containing protein 7-like [*Sphaeramia orbicularis*]	38.9	38.9	26%	0.019	73%	XP_029999321.1
transmembrane emp24 domain-containing protein 7-like [*Takifugu rubripes*]	38.9	38.9	26%	0.019	73%	XP_003974884.1
transmembrane emp24 domain-containing protein 7-like [*Seriola dumerili*]	38.9	38.9	26%	0.019	73%	XP_022613501.1
transmembrane emp24 domain-containing protein 7-like [*Cynoglossus semilaevis*]	38.9	38.9	26%	0.020	73%	XP_008335949.1
transmembrane emp24 domain-containing protein 7-like [*Chanos chanos*]	38.5	38.5	26%	0.022	73%	XP_030631937.1
transmembrane emp24 domain-containing protein 7-like [*Tachysurus fulvidraco*]	35.4	35.4	26%	0.280	68%	XP_027022590.1
transmembrane emp24 domain-containing protein 7 precursor [*Danio rerio*]	40.4	40.4	29%	0.005	67%	NP_001300637.1
transmembrane emp24 domain-containing protein 7 precursor [*Oncorhynchus mykiss*]	39.7	39.7	26%	0.010	73%	ACO08146.1

transmembrane emp24 domain-containing protein 7 precursor [*Esox lucius*]	39.3	39.3	26%	0.012	73%	NP_001290769.1
transmembrane emp24 domain-containing protein 7 [*Anabarilius grahami*]	41.2	41.2	77%	0.003	38%	ROL53726.1
transmembrane emp24 domain-containing protein 7-like isoform X1 [*Anabas testudineus*]	40.8	40.8	27%	0.004	74%	XP_026199701.1
transmembrane emp24 domain-containing protein 7-like isoform X2 [*Anabas testudineus*]	40.4	40.4	27%	0.005	74%	XP_026199702.1
transmembrane emp24 domain-containing protein 7-like [*Pangasianodon hypophthalmus*]	39.7	39.7	26%	0.008	73%	XP_026781058.1
transmembrane emp24 domain-containing protein 7 [*Oryzias latipes*]	39.7	39.7	26%	0.009	73%	XP_004072289.1
transmembrane emp24 domain-containing protein 7 [*Salvelinus alpinus*]	39.7	39.7	26%	0.009	73%	XP_023834706.1
transmembrane emp24 domain-containing protein 7 [*Cyclopterus lumpus*]	39.7	39.7	26%	0.009	73%	XP_034396819.1
transmembrane emp24 domain-containing protein 7 [*Liparis tanakae*]	39.7	39.7	26%	0.010	73%	TNN55930.1
transmembrane emp24 domain-containing protein 7 [*Pseudochaenichthys georgianus*]	39.7	39.7	26%	0.010	73%	XP_033947282.1
transmembrane emp24 domain-containing protein 7 [*Astyanax mexicanus*]	39.3	39.3	26%	0.011	73%	XP_007260848.1
transmembrane emp24 domain-containing protein 7 isoform X1 [*Maylandia zebra*]	39.7	39.7	26%	0.011	73%	XP_004547316.1
transmembrane emp24 domain-containing protein 7 [*Larimichthys crocea*]	39.3	39.3	26%	0.013	73%	XP_010746796.1
transmembrane emp24 domain-containing protein 7 isoform X2 [*Maylandia zebra*]	39.3	39.3	26%	0.014	73%	XP_004547317.1
transmembrane emp24 domain-containing protein 7 [*Oreochromis niloticus*]	39.3	39.3	26%	0.014	73%	XP_003446109.1

		transmembrane emp24 domain-containing protein 7 [*Scleropages formosus*]	38.9	38.9	26%	0.016	73%	XP_029115340.1	
		transmembrane emp24 domain-containing protein 7 [*Epinephelus lanceolatus*]	38.9	38.9	26%	0.017	73%	XP_033476377.1	
		transmembrane emp24 domain-containing protein 7 [*Scophthalmus maximus*]	38.9	38.9	26%	0.017	73%	AWP07242.1	
		transmembrane emp24 domain-containing protein 7 isoform X2 [*Fundulus heteroclitus*]	38.9	38.9	26%	0.018	73%	XP_012712633.1	
		transmembrane emp24 domain-containing protein 7 [*Periophthalmus magnuspinnatus*]	38.9	38.9	26%	0.018	73%	XP_033827945.1	
		transmembrane emp24 domain-containing protein 7 [*Thalassophryne amazonica*]	38.9	38.9	26%	0.018	73%	XP_034026363.1	
		transmembrane emp24 domain-containing protein 7 isoform X1 [*Fundulus heteroclitus*]	38.9	38.9	26%	0.019	73%	XP_021168982.1	
		transmembrane emp24 domain-containing protein 7 [*Paramormyrops kingsleyae*]	37.0	37.0	26%	0.056	68%	XP_023694715.1	
Mmer-EST_13.1	ERR048979.44781	Inactive phospholipase D5 [*Fundulus heteroclitus*]	31.6	31.6	52%	2.1	45%	JAR52186.1	ACCATGTAAAATAAAGACACACGCCCGCACAGTCACAG<br>GTACACACTGACACT**CACACACACACACACACACA**TTT<br>TGCACTACAAAGGACAAACGCACTCACGCTTTGCTACA<br>TGTTCACACAAGAAATCCTCCTTCGCCTTCTATGATTTC<br>TTTTGAACAAATGTCAACGTTCATTTGGAGATCTTTCAA<br>TGCACATATGAATGGCTTTGTCATGGGATTTGTTACG
Mmer-EST_10.2	ERR048978.23682	Kinesin-like protein KIF2A [*Danio rerio*]	34.7	34.7	25%	0.054	62%	XP_021334897.1	GTATCTGTCTTGGAAACATGGTGAACTTAATCTGAATAA<br>ATGTCAATACCCAAATGCCAGCAAATCGATTAGACAAT<br>AAGTTACTACCAATCCCAAAAGTAACCACAGAACACAT<br>TTGGTTTATTTTGGGCACAGATTTATGACT**TGTGTGTGT**<br>**GTGTGTG**TCTGTGTAGTACATCTGGTAACGGTTCAGGA<br>CATTAAGTCAGCTGATGGTCCGGCGCTGACGTGTGAAT<br>CCCTAAACAGATTT
Mmer-EST_13.3	ERR048980.40371	NPM							TTTCCAGAACATTTTCAGAAAATACGGCACTTAATGAA<br>AGCGTTGGTGTTGAAGCAACGTGATGATCTCCATGTTTC<br>TTTTCAGTCGCATTGGTTTATGTTTATCACC**TGTTGTTG**<br>**TTGTTGTTGT**TGGTCGGAGAGCGCTGCCCTCCTGCTGA<br>GCTGGGAATGAACAAATGAATGCTTTGAAGG**TTTTTTT**

										**TT**A**TTTTTTTTT**GCGCATGTAAATAGCGCACTGGCTGCTCCATAGGGTGGAGGTGGACAA
Tetraplex 2	Mmer-EST_9.1	ERR048978.69936	Hypothetical protein [*Anguilla anguilla*]	37.0	37.0	45%	0.005	56%	JAH98183.1	CCCCTGGTCCCAAATAAATCCTGGTTTAAAGTTGAGAACCCTTCCCCTTTGTAAACAAGCTGCGTTTCTCGCCACCTCCTCACT**CACACACACACA**AACATTTGGAAGCTTAAGGACATATATCCAAGACATCTATATAAAACTCTAGATGTGCAATAAAATAACTTTTGTAAAACTTGATGGGCACATACGTACAAAGGGCCTACACATTAAATCAAGTAGCACCATTCAATCCTAAATTCAACACTGTCAGTGC
	Mmer-EST_14.2	ERR048980.18528	Ubiquitin specific peptidase 40 [*Nothobranchius furzeri*]	31.6	31.6	19%	2.8	65%	SBS45312.1	GGGGGGAGGGAGGAAGAGGAATAGAAAGCGTGATACTTTTTAATTCCCACTGTTCGGCTGTGAACCGCGACCTCGTCTCCTCCGCCGACGCCGGCTATACCGTGACACGCGC**GTGTGTGTGTGTGTGTGTGT**TTCTGTACAGTTACAGCGTAGCACCAGAAGCTGTAGGGTGGGCTTTTCGCTCTCGTCCATCTTGCATGGTTTCCGAGTCAAACGGGGGGTGAGTCACGCCAGAATGGTGTGTGACTCACTTGGTG
	Mmer-EST_1.3	ERR048979.2798	NPM							GGGGGGCGACGCGCGCCCGCTTTATTTGATCTCCGTGGTAGATGGCTCATCTAATGCACTTCTTTGTGTTGAATCAGTCGGCTCCATT**AATAATAATAATAATAATAATAATAATAATAATAATAAT**GTATGCTATATTTAACTTATTTGTCGGCAGCAATTTTAAAAACGCCCAGCAGTCGA
	Mmer-EST_14.4	ERR048980.28127	NPM							CTGCGCACCGCCGTTCGCCAGGACGGCACCGGTGTCACATCTGGTTAGACGAGTCCTGCAAGCAGAACTACAAAACGGCAAACAGAGCTGTCAACGCAGCCGGAGAGGAGGAGGAGGAAGTGGGAATGCAACAGCAAGGAATGTAAACCGCACTTAAGAGCTTTGAAACTGGGGTG**AGAGAGAGAGAGA**AGGAATGAGGAAGAAGTAATGTTAACCGGCAACACCTCTTGTCTCCCGCCTGTCTCTCG
Tetraplex 3	Mmer-EST_8.1[†]	ERR048977.3541	MTSS1-like protein [*Channa argus*]	61.2	61.2	51%	2.00E-10	79%	KAF3689057.1	CGTTGGTACACTTTGCTACTCAGCTAGTCAGG**ACACACACACACACACACACACACACAC**TTGCTCGTGCACGCACGCAGCAGTGGCAGCGGTCTAGCCAAGGATCCTGGGCGCCGAGCGGTCGTCGGTCGCCACCTTGCGGAGCCTCACGCCACGCCGGATGGACACCAGCACGTCCACTTCCTCTCTGGCAGAGGAAGAGAAGGGCTCCGTGGGGGAAGGACGCGG
			MTSS1-like protein isoform X3 [*Perca flavescens*]	57.0	57.0	51%	6.00E-09	74%	XP_028440813.1	
			MTSS1-like protein isoform X2 [*Parambassis ranga*]	56.6	56.6	48%	9.00E-09	73%	XP_028264257.1	

MTSS1-like protein [*Larimichthys crocea*]	54.3	54.3	52%	1.00E-08	70%	TMS05842.1
MTSS1-like protein isoform X1 [*Fundulus heteroclitus*]	54.3	54.3	51%	6.00E-08	74%	XP_012715788.1
MTSS1-like protein isoform X1 [*Oncorhynchus tshawytscha*]	54.3	54.3	47%	6.00E-08	75%	XP_024235587.1
MTSS1-like protein isoform X1 [*Oreochromis niloticus*]	54.3	54.3	44%	6.00E-08	76%	XP_003447182.1
MTSS1-like protein isoform X3 [*Oryzias latipes*]	53.1	53.1	48%	1.00E-07	65%	XP_023811886.1
MTSS1-like protein isoform X1 [*Boleophthalmus pectinirostris*]	52.8	52.8	56%	2.00E-07	68%	XP_020786751.1
MTSS1-like protein isoform X1 [*Cynoglossus semilaevis*]	53.1	53.1	42%	2.00E-07	78%	XP_008310587.1
MTSS1-like protein isoform X1 [*Kryptolebias marmoratus*]	52.8	52.8	48%	2.00E-07	65%	XP_017274520.1
MTSS1-like protein isoform X1 [*Maylandia zebra*]	52.8	52.8	44%	2.00E-07	71%	XP_004570835.1
MTSS1-like protein isoform X1 [*Oncorhynchus mykiss*]	52.4	52.4	47%	3.00E-07	72%	XP_021430036.1
MTSS1-like protein isoform X1 [*Salvelinus alpinus*]	52.0	52.0	48%	4.00E-07	70%	XP_023827760.1
MTSS1-like protein isoform X1 [*Seriola dumerili*]	51.2	51.2	51%	7.00E-07	69%	XP_022610317.1
MTSS1-like protein isoform X1 [*Seriola lalandi dorsalis*]	51.2	51.2	51%	7.00E-07	69%	XP_023271283.1
MTSS1-like protein isoform X1 [*Acanthochromis polyacanthus*]	50.8	50.8	50%	1.00E-06	61%	XP_022045320.1
MTSS1-like protein isoform X1 [*Amphiprion ocellaris*]	50.4	50.4	50%	1.00E-06	61%	XP_023155739.1
MTSS1-like protein isoform X1 [*Xiphophorus couchianus*]	50.4	50.4	44%	1.00E-06	71%	XP_027854880.1

MTSS1-like protein isoform X1 [*Xiphophorus maculatus*]	50.4	50.4	44%	1.00E-06	71%	XP_014325801.2
MTSS1-like protein isoform X1 [*Oryzias melastigma*]	49.7	49.7	53%	2.00E-06	59%	XP_024137089.1
MTSS1-like protein isoform X2 [*Monopterus albus*]	49.3	49.3	44%	3.00E-06	68%	XP_020458629.1
MTSS1-like protein isoform X1 [*Danio rerio*]	48.9	48.9	42%	5.00E-06	72%	XP_017212570.1
MTSS1-like protein [*Liparis tanakae*]	48.5	48.5	55%	6.00E-06	57%	TNN68000.1
MTSS1-like protein isoform X1 [*Gouania willdenowi*]	48.5	48.5	44%	6.00E-06	71%	XP_028305884.1
MTSS1 isoform X4 [*Labeo rohita*]	48.5	48.5	53%	7.00E-06	53%	RXN11023.1
MTSS1-like protein isoform X1 [*Astyanax mexicanus*]	47.8	47.8	53%	1.00E-05	53%	XP_015461633.1
MTSS1-like protein [*Bagarius yarrelli*]	47.0	47.0	40%	2.00E-05	71%	TSK19988.1
MTSS1-like protein [*Electrophorus electricus*]	46.6	46.6	39%	3.00E-05	73%	XP_026860561.1
MTSS1-like protein isoform X1 [*Tachysurus fulvidraco*]	46.6	46.6	39%	3.00E-05	73%	XP_026992805.1
MTSS1-like protein [*Triplophysa tibetana*]	46.2	46.2	38%	4.00E-05	76%	KAA0723585.1
MTSS1-like protein [*Collichthys lucidus*]	45.8	45.8	53%	5.00E-05	54%	TKS70314.1
MTSS1-like protein isoform X1 [*Paramormyrops kingsleyae*]	45.8	45.8	38%	7.00E-05	76%	XP_023678497.1
MTSS1-like protein isoform X7 [*Carassius auratus*]	45.8	45.8	40%	7.00E-05	68%	XP_026114869.1
Protein MTSS 1 isoform X1 [*Myripristis murdjan*]	44.7	44.7	53%	2.00E-04	59%	XP_029919987.1

Protein MTSS 1-like isoform X1 [*Archocentrus centrarchus*]	44.3	44.3	42%	2.00E-04	59%	XP_030605358.1
MTSS1-like protein [*Anabarilius grahami*]	43.5	43.5	39%	3.00E-04	70%	ROL43599.1
Protein MTSS 1 isoform X1 [Salmo trutta]	43.5	43.5	38%	4.00E-04	66%	XP_029587687.1
Protein MTSS 1-like [Sphaeramia orbicularis]	43.5	43.5	36%	4.00E-04	71%	XP_029985766.1
Protein MTSS 1 isoform X10 [*Takifugu rubripes*]	43.1	43.1	51%	5.00E-04	54%	XP_029694179.1
MTSS1-like protein isoform X1 [*Astatotilapia calliptera*]	43.1	43.1	44%	5.00E-04	59%	XP_026031589.1
Protein MTSS 1 isoform X2 [*Oncorhynchus kisutch*]	42.7	42.7	34%	6.00E-04	69%	XP_031649443.1
Protein MTSS 1 isoform X2 [*Oncorhynchus nerka*]	42.7	42.7	34%	6.00E-04	69%	XP_029514111.1
Metastasis suppressor protein 1 [*Takifugu flavidus*]	43.1	43.1	51%	6.00E-04	54%	TWW79466.1
Protein MTSS 1 isoform X1 [*Esox lucius*]	42.4	42.4	34%	0.001	69%	XP_012988144.1
Protein MTSS 1-like isoform X1 [*Oreochromis aureus*]	42.0	42.0	40%	0.001	55%	XP_031607691.1
Protein MTSS 1-like isoform X1 [*Periophthalmus magnuspinnatus*]	42.0	42.0	55%	0.001	45%	XP_033836399.1
Protein MTSS 1-like isoform X1 [*Scleropages formosus*]	42.4	42.4	51%	0.001	54%	XP_018582727.1
Protein MTSS 1-like [*Thalassophryne amazonica*]	41.6	41.6	35%	0.002	63%	XP_034031131.1
Protein MTSS 1-like isoform X1 [*Xiphophorus hellerii*]	41.6	41.6	47%	0.002	46%	XP_032414486.1
Protein MTSS 1 [*Salarias fasciatus*]	41.2	41.2	50%	0.003	55%	XP_029976858.1

Protein MTSS 1 isoform X1 [*Mastacembelus armatus*]	40.8	40.8	32%	0.003	72%	XP_026156241.1
Protein MTSS 1 isoform X1 [*Anabas testudineus*]	40.4	40.4	32%	0.004	68%	XP_026205192.1
Protein MTSS 1 isoform X1 [*Anarrhichthys ocellatus*]	40.8	40.8	32%	0.004	72%	XP_031734352.1
Protein MTSS 1 isoform X1 [*Betta splendens*]	40.8	40.8	32%	0.004	76%	XP_029023590.1
Protein MTSS 1 isoform X1 [*Clupea harengus*]	40.4	40.4	42%	0.005	56%	XP_031441805.1
Protein MTSS 1-like isoform X1 [*Trematomus bernacchii*]	39.7	39.7	34%	0.009	65%	XP_033980648.1
Protein MTSS 1 [*Cottoperca gobio*]	39.7	39.7	34%	0.010	65%	XP_029298934.1
Protein MTSS 1-like [*Cyclopterus lumpus*]	39.7	39.7	55%	0.010	52%	XP_034401059.1
Protein MTSS 1-like isoform X1 [*Epinephelus lanceolatus*]	39.7	39.7	32%	0.010	68%	XP_033493401.1
Protein MTSS 1-like isoform X5 [*Chanos chanos*]	39.7	39.7	31%	0.010	67%	XP_030637832.1
Protein MTSS 1 isoform X1 [*Sander lucioperca*]	39.3	39.3	32%	0.011	68%	XP_031163545.1
Protein MTSS 1-like isoform X1 [*Labrus bergylta*]	39.3	39.3	35%	0.012	59%	XP_020490214.1
Protein MTSS 1-like isoform X4 [*Gadus morhua*]	38.9	38.9	47%	0.018	47%	XP_030227262.1
Protein MTSS 1-like isoform X1 [*Echeneis naucrates*]	38.5	38.5	32%	0.022	68%	XP_029370024.1
Protein MTSS 1-like isoform X2 [*Etheostoma spectabile*]	38.1	38.1	32%	0.030	60%	XP_032373927.1
Protein MTSS 1-like isoform X1 [*Sparus aurata*]	38.1	38.1	32%	0.034	60%	XP_030250116.1

Protein MTSS 1-like isoform X1 [*Gymnodraco acuticeps*]	36.6	36.6	32%	0.100	56%	XP_034064480.1
Protein MTSS 1-like isoform X6 [*Denticeps clupeoides*]	36.2	36.2	31%	0.150	58%	XP_028841159.1
Protein MTSS 1-like isoform X1 [*Pseudochaenichthys georgianus*]	36.2	36.2	32%	0.170	56%	XP_033958033.1
Protein MTSS 1 isoform X2 [*Pangasianodon hypophthalmus*]	35.0	35.0	34%	0.370	62%	XP_034163490.1
Protein MTSS 2-like isoform X1 [*Gadus morhua*]	67.4	67.4	52%	1.00E-12	82%	XP_030222100.1
Protein MTSS 2-like isoform X1 [*Sander lucioperca*]	57.4	57.4	52%	5.00E-09	73%	XP_031139049.1
Protein MTSS 2-like isoform X2 [*Sphaeramia orbicularis*]	56.6	56.6	43%	9.00E-09	82%	XP_029992579.1
Protein MTSS 2-like isoform X1 [*Archocentrus centrarchus*]	55.5	55.5	48%	2.00E-08	73%	XP_030588057.1
Protein MTSS 2-like isoform X1 [*Betta splendens*]	55.5	55.5	46%	2.00E-08	74%	XP_029009980.1
Protein MTSS 2-like isoform X1 [*Echeneis naucrates*]	55.1	55.1	44%	3.00E-08	76%	XP_029378151.1
Protein MTSS 2-like isoform X1 [*Etheostoma spectabile*]	55.1	55.1	44%	3.00E-08	76%	XP_032379715.1
Protein MTSS 2-like isoform X1 [*Mastacembelus armatus*]	55.1	55.1	50%	3.00E-08	68%	XP_026168203.1
Protein MTSS 2-like isoform X1 [*Labrus bergylta*]	54.7	54.7	52%	5.00E-08	68%	XP_020495426.1
Protein MTSS 2-like isoform X1 [*Oreochromis aureus*]	54.3	54.3	44%	6.00E-08	76%	XP_031585857.1
Protein MTSS 2-like isoform X1 [*Myripristis murdjan*]	53.9	53.9	55%	8.00E-08	74%	XP_029910332.1
Protein MTSS 2-like isoform X3 [*Cottoperca gobio*]	53.1	53.1	56%	1.00E-07	63%	XP_029289404.1

Protein MTSS 2-like isoform X3 [*Salarias fasciatus*]	53.5	53.5	53%	1.00E-07	68%	XP_029951351.1
Protein MTSS 2-like isoform X1 [*Oncorhynchus nerka*]	52.8	52.8	47%	2.00E-07	72%	XP_029523587.1
Protein MTSS 2-like isoform X1 [*Anarrhichthys ocellatus*]	52.4	52.4	51%	3.00E-07	67%	XP_031712059.1
Protein MTSS 2-like isoform X1 [*Salmo trutta*]	52.0	52.0	46%	3.00E-07	74%	XP_029614707.1
Protein MTSS 2 isoform X1 [*Oncorhynchus kisutch*]	52.0	52.0	48%	4.00E-07	70%	XP_031678809.1
Protein MTSS 2 isoform X3 [*Takifugu rubripes*]	49.3	49.3	48%	3.00E-06	69%	XP_029697449.1
Protein MTSS 2 isoform X1 [*Esox lucius*]	47.0	47.0	35%	2.00E-05	81%	XP_019910660.1
Protein MTSS 2 isoform X4 [*Epinephelus lanceolatus*]	47.0	47.0	56%	2.00E-05	53%	XP_033484457.1
Protein MTSS 2 isoform X1 [*Anabas testudineus*]	46.6	46.6	53%	3.00E-05	54%	XP_026233593.1
Protein MTSS 2 isoform X1 [*Clupea harengus*]	46.6	46.6	50%	3.00E-05	61%	XP_031425490.1
Protein MTSS 2 isoform X1 [*Pangasianodon hypophthalmus*]	47.0	47.0	39%	3.00E-05	73%	XP_026793306.1
Protein MTSS 2-like isoform X1 [*Sparus aurata*]	46.6	46.6	53%	3.00E-05	54%	XP_030271134.1
Protein MTSS 2-like isoform X4 [*Xiphophorus hellerii*]	46.6	46.6	52%	3.00E-05	55%	XP_032415837.1
Protein MTSS 2 [*Chanos chanos*]	46.2	46.2	39%	4.00E-05	73%	XP_030620864.1
Protein MTSS 2 isoform X1 [*Denticeps clupeoides*]	45.8	45.8	38%	6.00E-05	76%	XP_028811704.1
Protein MTSS 2 isoform X5 [*Scleropages formosus*]	45.8	45.8	38%	6.00E-05	76%	XP_029109165.1

		Protein MTSS 2 isoform X1 [*Cyclopterus lumpus*]	45.4	45.4	56%	7.00E-05	53%	XP_034418560.1	
		Protein MTSS 2 isoform X1 [*Pseudochaenichthys georgianus*]	44.3	44.3	56%	2.00E-04	54%	XP_033960734.1	
		Protein MTSS 2-like isoform X1 [*Trematomus bernacchii*]	44.3	44.3	56%	2.00E-04	54%	XP_033972491.1	
		Protein MTSS 2-like isoform X1 [*Gymnodraco acuticeps*]	43.9	43.9	52%	3.00E-04	55%	XP_034087772.1	
		Protein MTSS 2 isoform X1 [*Thalassophryne amazonica*]	43.5	43.5	50%	4.00E-04	55%	XP_034039392.1	
		Protein MTSS 2 isoform X1 [*Periophthalmus magnuspinnatus*]	43.1	43.1	52%	5.00E-04	53%	XP_033845144.1	
Mmer-EST_8.2‡	ERR048976.6906	protein scribble homolog isoform X1 [*Monopterus albus*]	49.7	49.7	34%	3.00E-06	82%	XP_020473123.1	AGGAGGTGGATGCGGGGGTGTTGGAGGACATGGCGCTGTACAGCAACAAACGCAAACTGAGAGCGGGCCGCCACAGCCTGGAGGCGGCGGTCCCCACATAACACCG**ACACACACACACACACAC**AGAACCCGCGCCGACCCGCGAGGACCGCCTGATGTATGGAAACCACTGCGGGAAAACAATGGAGACGATCCAACGACCATCCGAACTTTTCACTAGTAGTCACTTCCTGCTTGCGTCTAGG
		protein scribble homolog isoform X10 [*Tachysurus fulvidraco*]	48.5	48.5	30%	9.00E-06	88%	XP_027025169.1	
		protein scribble homolog isoform X1 [*Carassius auratus*]	47.8	47.8	30%	1.00E-05	88%	XP_026071392.1	
		protein scribble homolog isoform X11 [*Gadus morhua*]	48.1	48.1	37%	1.00E-05	77%	XP_030204161.1	
		protein scribble homolog isoform X13 [*Xiphophorus hellerii*]	46.6	46.6	30%	3.00E-05	88%	XP_032406893.1	
		protein scribble homolog isoform X7 [*Xiphophorus maculatus*]	46.6	46.6	30%	3.00E-05	88%	XP_023182532.1	
		protein scribble homolog isoform X1 [*Amphiprion ocellaris*]	46.6	46.6	30%	4.00E-05	88%	XP_023128465.1	
		protein scribble homolog isoform X1 [*Xiphophorus couchianus*]	46.6	46.6	30%	4.00E-05	88%	XP_027860506.1	

protein scribble homolog [*Scleropages formosus*]	45.4	45.4	34%	9.00E-05	75%	XP_029107910.1
protein scribble homolog [*Boleophthalmus pectinirostris*]	45.1	45.1	30%	1.00E-04	84%	XP_020791390.1
protein scribble homolog [*Dicentrarchus labrax*]	45.1	45.1	30%	1.00E-04	84%	CBN82113.1
protein scribble homolog [*Oreochromis aureus*]	45.1	45.1	30%	1.00E-04	84%	XP_031607551.1
protein scribble homolog [*Oryzias melastigma*]	45.4	45.4	31%	1.00E-04	81%	XP_024119694.1
protein scribble homolog isoform X1 [*Acanthochromis polyacanthus*]	45.1	45.1	30%	1.00E-04	84%	XP_022055054.1
protein scribble homolog isoform X1 [*Anabas testudineus*]	45.1	45.1	30%	1.00E-04	84%	XP_026217627.1
protein scribble homolog isoform X1 [*Archocentrus centrarchus*]	45.1	45.1	30%	1.00E-04	84%	XP_030612518.1
protein scribble homolog isoform X1 [*Astatotilapia calliptera*]	45.1	45.1	30%	1.00E-04	84%	XP_026035712.1
protein scribble homolog isoform X1 [*Chanos chanos*]	45.1	45.1	30%	1.00E-04	84%	XP_030635367.1
protein scribble homolog isoform X1 [*Epinephelus lanceolatus*]	45.1	45.1	30%	1.00E-04	84%	XP_033494342.1
protein scribble homolog isoform X1 [*Gouania willdenowi*]	45.1	45.1	30%	1.00E-04	84%	XP_028289660.1
protein scribble homolog isoform X1 [*Gymnodraco acuticeps*]	45.1	45.1	30%	1.00E-04	84%	XP_034070061.1
protein scribble homolog isoform X1 [*Kryptolebias marmoratus*]	45.1	45.1	30%	1.00E-04	84%	XP_017262004.1
protein scribble homolog isoform X1 [*Labrus bergylta*]	45.1	45.1	30%	1.00E-04	84%	XP_020489301.1
protein scribble homolog isoform X1 [*Laramichthys crocea*]	45.1	45.1	30%	1.00E-04	84%	XP_027130097.1

protein scribble homolog isoform X1 [*Mastacembelus armatus*]	45.1	45.1	30%	1.00E-04	84%	XP_026148266.1
protein scribble homolog isoform X1 [*Maylandia zebra*]	45.1	45.1	30%	1.00E-04	84%	XP_012774778.2
protein scribble homolog isoform X1 [*Myripristis murdjan*]	45.1	45.1	30%	1.00E-04	84%	XP_029935660.1
protein scribble homolog isoform X1 [*Oreochromis niloticus*]	45.1	45.1	30%	1.00E-04	84%	XP_025765932.1
protein scribble homolog isoform X1 [*Pangasianodon hypophthalmus*]	45.1	45.1	30%	1.00E-04	84%	XP_034159795.1
protein scribble homolog isoform X1 [*Parambassis ranga*]	45.1	45.1	30%	1.00E-04	84%	XP_028288809.1
protein scribble homolog isoform X1 [*Paramormyrops kingsleyae*]	45.1	45.1	34%	1.00E-04	75%	XP_023688580.1
protein scribble homolog isoform X1 [*Seriola dumerili*]	45.1	45.1	30%	1.00E-04	84%	XP_022622774.1
protein scribble homolog isoform X1 [*Seriola lalandi dorsalis*]	45.1	45.1	30%	1.00E-04	84%	XP_023264122.1
protein scribble homolog isoform X1 [*Sparus aurata*]	45.1	45.1	30%	1.00E-04	84%	XP_030254688.1
protein scribble homolog isoform X1 [*Sphaeramia orbicularis*]	45.1	45.1	30%	1.00E-04	84%	XP_029979702.1
protein scribble homolog isoform X1 [*Takifugu rubripes*]	45.1	45.1	30%	1.00E-04	84%	XP_029698381.1
protein scribble homolog isoform X1 [*Trematomus bernacchii*]	45.1	45.1	30%	1.00E-04	84%	XP_034004346.1
protein scribble homolog isoform X10 [*Anarrhichthys ocellatus*]	45.1	45.1	30%	1.00E-04	84%	XP_031705692.1
protein scribble –like protein [*Takifugu flavidus*]	45.1	45.1	30%	1.00E-04	84%	TWW70794.1
protein scribble –like protein Scribble1 [*Channa argus*]	45.1	45.1	30%	1.00E-04	84%	KAF3703730.1

protein scribble homolog [*Danio rerio*]	44.7	44.7	30%	2.00E-04	84%	NP_001007176.1
protein scribble homolog [*Fundulus heteroclitus*]	45.1	45.1	30%	2.00E-04	84%	XP_021167343.1
protein scribble homolog isoform X1 [*Esox lucius*]	45.1	45.1	30%	2.00E-04	84%	XP_019897341.2
protein scribble –like protein [*Triplophysa tibetana*]	44.7	44..7	30%	2.00E-04	84%	KAA0723516.1
protein scribble homolog isoform X1 [*Salarias fasciatus*]	44.3	44..3	30%	3.00E-04	80%	XP_029955840.1
protein scribble homolog isoform X2 [*Clupea harengus*]	43.9	43.9	36%	3.00E-04	70%	XP_031439491.1
protein scribble-like protein [*Perca flavescens*]	43.9	43.9	31%	3.00E-04	77%	ADX97060.1
protein scribble homolog isoform X13 [*Etheostoma spectabile*]	43.5	43.5	30%	4.00E-04	80%	XP_032359907.1
protein scribble homolog isoform X1 [*Sander lucioperca*]	43.5	43.5	30%	5.00E-04	80%	XP_031148678.1
protein scribble homolog isoform X1 [*Astyanax mexicanus*]	43.1	43.1	30%	6.00E-04	80%	XP_022533770.1
protein scribble homolog isoform X15 [*Denticeps clupeoides*]	43.1	43.1	30%	6.00E-04	80%	XP_028815868.1
protein scribble homolog [*Electrophorus electricus*]	43.1	43.1	31%	7.00E-04	77%	XP_026875185.1
protein scribble homolog isoform X7 [*Oncorhynchus kisutch*]	43.1	43.1	31%	7.00E-04	77%	XP_031691911.1
protein scribble homolog isoform X8 [*Salvelinus alpinus*]	43.1	43.1	31%	7.00E-04	77%	XP_023828481.1
protein scribble homolog [*Oncorhynchus mykiss*]	43.1	43.1	31%	8.00E-04	77%	XP_021418548.1
protein scribble homolog [*Oncorhynchus tshawytscha*]	42.7	42.7	31%	8.00E-04	77%	XP_024248149.1

			protein scribble homolog isoform X1 [*Salmo trutta*]	42.7	42.7	31%	8.00E-04	77%	XP_029611790.1	
			protein scribble homolog isoform X1 [*Oryzias latipes*]	42.7	42.7	30%	9.00E-04	80%	XP_023805912.1	
			protein scribble homolog isoform X1 [*Pseudochaenichthys georgianus*]	42.4	42.4	30%	0.001	80%	XP_033964577.1	
			protein scribble homolog isoform X10 [*Oncorhynchus nerka*]	34.7	34.7	28%	0.640	70%	XP_029482776.1	
	Mmer-EST_8.3	ERR048977.54842	RASF3 [*Poeciliopsis prolifica*]	29.6	29.6	32%	7.6	57%	JAO86479.1	GCTGGCGCCCCCTGCCGCCGGCCTCGGCCCTCGCACGGTCATTGGTCGGCGGATGCTCCGGAGAGGCTCCTCCACTACCCACCTGATGGGGCGTCTGCTGAAGTTTTAAGGTGGTGATGGTGG**TGCTGCTGGTGCTGCTGCTGCTGCTGC**TCACTGGCCGGTTCTGTCATCCTGGTGATGGTGGCAGATGATGGTT
	Mmer-EST_8.4	ERR048980.23857	NPM							TGCAGCATTACTTGTCGCCTCGCCTTTTGCGACGGCTACATTCTGAGAAATCTCGATTTCCATGCAGTCTTTTGATACTGATAATCCCATCCCACTTTAATTCAGCAGACCATTCGGTAAGTCAGTCGGTCA**TTCATTCATTCATTCATTCATTCATTCA**CAGTACGTTTTGTGGACCTAACCGATGTTCATTTTCCTAAACTGTTGGGTTAATTGTGTTGTGTGTTGTCCACAGCT
Tetraplex 4	Mmer-EST_3.1	ERR048979.11233	NPM							GGGCTATAATGGCATCGCTATGCGTTATGGGAGAACTGTG**ATATATATATAT**AAATAAATATAACAGTCAAATTAGGATACATTCTTGGTCCATCTTAAACACTAGGTCAATATTCTAAATAGGAATAAACAACAAATTTGACCATCACCCCGTTTTAACACATTAGGGTGACACAAATAACTGATCATATTAAGTCTACAAATTTGGATTTGAACCTCTGGGGCGGGGGGCTGTCCATGGGAAGTGGTAGAT
	Mmer-EST_6.3	ERR048978.33848	NPM							CAATAGGCATGTATTGCACACACTACTTGTTGCCGCTGTCTAGCTTAGCCCGACGCTAACTGCTGTAGCGCACTTAAGTAGTGTAGTGTTGCCTTGGTAACTAACCCTGCCTCCCTCTTCCT**TCCTCCTCCTCCTCCTCCTCCT**ATCCCGGTGCACGGTTTCTTCTTTGATGCTAGGGTTAAACCAGACCCCTCCCCCTCCTCNNCTCCTCCTCCTCCCTCCCTCCT
	Mmer-EST_6.4	ERR048981.36397	NPM							GTTGGGCAACTTTCCCTTTAGTTTGCCCGGGAACGACG**CCGCCGCCGCCGCCG**CTCGCGACTCCACGCTGACGCAGACGACGTCTCCCGAACTTTCTTGGGGTCGGAACAACGT

				GTCGTCGGGAAATGGGTTCGCCTCGCGCCACGCTCCCC<br>GCACATAC
	Mmer-EST_9.4	ERR048980.47967	NPM	CCCCGATCAACCCGGGAGAGTTGGCAACCATAGTCCCT<br>ATGCTAGTAAACAAAAAAAAACACACAACCCAATTGTA<br>GCTGTATCTGCTTTAATGAA**GTATGTATGTATGTATGT**<br>**ATGTATGTATGTATGTATGTATGTAT**GTCATTTAAACC<br>ATGGATTACAGAAACACATTGAACATATAATACTTGAC<br>ATACACGCAATACTCTCTCTCTATACTGAACCCGTCCTG<br>AAAAGCTG
Tetraplex 5	Mmer-EST_2.1	ERR048977.35422	NPM	CGGACGGCAACCACAACGATATCCTTTCCTGTGGCTCAT<br>CTTTCA**CGGCGGCGGCGGCGGCGGCGGCGGC**GCTCG<br>CCGTGTAGCGCTGGAGTCACATGACACGGCCAAAACCT<br>TTCCTCTATTATGTTATCAACAACACCTTCTTCCAATCTT<br>TTCCACGTTTATTTTAAAATGGTCAACTACTGTATGTAT<br>TGTGGTGTGGTTGTGCACCGAGAGACCTCCCTAATTAGA<br>GACCAGCACCCAGGGGTCCTGCGAGGG
	Mmer-EST_2.2	ERR048980.37602	NPM	GAGACGATGACCGATGGCTTCCAGGTGAGCCACCCGAA<br>ACAACATTTCCAGACAATATTGCACTGCACTCTTTGTAC<br>AGTTCCATTGGTTGTGACAACTCAAGTCAATGTATTGAG<br>**TTATTATTATTATTATTA**TATGTTGTCCCCTACTGTTGT<br>GTTATAAGTTGTACCAGCAGGGTCTTCTGAGCCAAACTC<br>TGAACTCTGACCCCTCTCTCTCCTCCTCAGTTCGAGTAT<br>GGCAGCG
	Mmer-EST_4.3	ERR048978.72514	NPM	TGAATAAATATAATGGAGAAATTAATAAATATTATCTA<br>GGACTCTGACTGCTTGTGGAGAAGTAAGTCGACATTTA<br>AGATGCGTCTCAGGATAAGTTGGGTCTGCTAACAGTCTT<br>AGCGCTGTCGGCATGTAATGACCCCAA**TGTTGTTGTTG**<br>**TTGTTGTTGTTGTTGTTGT**CGTCTTACATTTCAATTCTG<br>CTTGTCCTGAATGTCAAGGCATTGATTTAAGCCGTACAT<br>TTAGTTTCTTTG
	Mmer-EST_2.4	ERR048982.60263	NPM	CATGCTGTTGTGTGCTGGAGGATCTTGGCTGAAAGACTA<br>GAGGTAAAATAAATAATAAAGGAATAAAGGACATGTCT<br>TTTAACACTTTCATTCCTGGAGGGCGATTCACGAAGAGC<br>AGTCAAAGTATAGAAATGTCA**GTTGTTGTTGTTGTTGT**<br>**T**ATAGCAACGGAAAACAACAGACCGGCTAAAGGTCGTG<br>AACTTTATCTTTCAACTTTATCTGTTTTCGTAGTATCGGG<br>TCACTATAGGTTATCAGCCG

† More than 1500 entries in NCBI databases: a single isoform per species is shown for this sequence.

‡More than 900 entries in NCBI databases: a single isoform per species is shown for this sequence.

Table S3. Statistical power (Pearson, chi-square method of POWSIM) of marker subsets (see Table 1) according to their cloning source (R, randomly cloned microsatellites; EST, transcriptome-diverted microsatellites) as well as to their selective classification after LOSITAN (directional selection) and BAYESCAN (balancing selection).

	**Atlantic *vs.* Mediterranean**		**Five regions**	
**Dataset**	**Power**	**Type I error**	**Power**	**Type I error**
All markers (n = 26)	1.000	0.033	1.000	0.026
EST-microsatellites (n = 21)	1.000	0.037	1.000	0.046
Random microsatellites (n = 5)	1.000	0.039	1.000	0.037
**Lositan**				
1 R directional marker (3b)	0.998	0.040	1.000	0.024
4 R neutral markers (29, 34, 9b, 20)	1.000	0.033	1.000	0.045
3 EST directional markers (9.1,14.2,6.4)	-	-	1.000	0.033
18 EST neutral markers	-	-	1.000	0.027
4 R + 21EST neutral markers	1.000	0.034	-	-
1 R + 3 EST directional markers	-	-	1.000	0.035
4 R + 18 EST neutral markers	-	-	1.000	0.033
**BayeScan**				
3 R balanced markers (34b, 9b, 20)	0.980	0.030	1.000	0.041
2 R neutral markers (29, 3b)	1.000	0.029	1.000	0.036
1 EST balanced marker (6.3)	0.196	0.032	-	-
20 ESTs neutral markers	1.000	0.020	-	-
3 ESTs balanced markers (11.2, 13.3, 6.3)	-	-	1.000	0.023
18 ESTs neutral markers	-	-	1.000	0.037
3 R + 1EST balanced markers	0.947	0.042	-	-
2 R + 20 EST neutral markers	1.000	0.033	-	-
3 R + 3 EST balanced markers	-	-	1.000	0.041
2 R + 18 EST neutral markers	-	-	1.000	0.046

Table S4. Source of microsatellites (R, anonymous or random loci; EST, EST-loci) and their selective classification after two F_{ST}-outlier based algorithms, i.e. LOSITAN (neutral polymorphism *vs.* selectively diversified polymorphism); BAYESCAN (neutral polymorphism *vs.* selectively balanced polymorphism). Superscripts ^{d} to ^{g} indicate putative aberrant *k*-pool scenarios regarding previous demographic and genetic data in this species.

			Level of population divergence					
			Between basins (Atlantic - Mediterranean)			Among five regions from the whole range[†]		
	**Microsatellite type**	**Polymorphism**	**No. Loci (code)**	**BAPS[‡]**	**GENELAND[‡]**	**No. Loci (code)**	**BAPS[§]**	**GENELAND[§]**
**LOSITAN**	Anonymous loci	Directional	1 R (3b)	$k = 2$	$k = 2$	1 R (3b)	$k = 2$	$k = 2$
		Neutral	4 R (29, 34b, 9b, 20)	$k = 1$	$k = 1$	4 R (29, 34b, 9b, 20)	$k = 1$	$k = 3$[¶]
	EST-loci	Directional	0	-	-	3 EST (9.1, 14.2, 6.4)	$k = 2$[¥]	$k = 2$[¥]
		Neutral	21 EST	$k = 1$	$k = 2$	18 EST	$k = 1$	$k = 2$
	All selective loci	Directional	1 R (3b)	$k = 2$	$k = 2$	4 (1 R + 3 EST)	$k = 2$[¥]	$k = 3$[»]
	All neutral loci	Neutral	25 (4 R + 21 EST)	$k = 1$	$k = 2$	22 (4 R+ 18 EST)	$k = 1$	$k = 1$
	All loci	Directional or neutral	26 (5 R+21 EST)	$k = 1$	$k = 2$	26 (5 R + 21 EST)	$k = 1$	$k = 2$
**BAYESCAN**	Anonymous loci	Balanced	3R (34b, 9b, 20)	$k = 1$	$k = 1$	3 R (34b, 9b, 20)	$k = 1$	$k = 3$[¶]
		Neutral	2R (3b, 29)	$k = 2$	$k = 2$	2 R (3b, 29)	$k = 2$	$k = 2$
	EST-loci	balanced	1 EST (6.3, no power)	$k = 1$	$k = 1$	3 EST (13.3, 11.2, 6.3)	$k = 1$	$k = 1$
		Neutral	20 EST	$k = 1$	$k = 2$	18 EST	$k = 1$	$k = 2$[¥]
	All selective loci	Balanced	4 (3R + 1EST)	$k = 1$	$k = 1$	6 (3 R + 3 EST)	$k = 1$	$k = 2$[ƫ]
	All neutral loci	Neutral	22 (2 R +20 EST)	$k = 1$	$k = 2$	20 (2 R + 18 EST)	$k = 1$	$k = 2$
	All loci	Balanced or neutral	26 (5 R + 21 EST)	k = 1	k = 2	26 (5 R + 21 EST)	k = 1	k = 2
	Only neutral loci	Neutral	21 neutral loci	$k = 1$	$k = 2$	16 neutral loci	$k = 1$	$k = 2$

[†]Regional sample codes (see Figure 2 for details): NAN, North Atlantic North; CAN, Central Atlantic North; SAN, South Atlantic North; MEW, Western Mediterranean Sea; MEC, Central Mediterranean Sea.

[‡]Gene pool *k* = 1 indicates a single hake genetic unit along the Atlantic and the Mediterranean, *k* = 2 indicates a significant genetic split between those basins.

[§]Gene pool *k* = 1 indicates a single hake genetic unit, *k* = 2 indicates a significant split between Atlantic regions (NAN, CAN, SAN) and Mediterranean regions (MEW, MEC).

[¶]Gene pool *k* = 3 indicates a significant split between 3 groups of regions: Atlantic North (NAN, CAN), Gulf of Cadiz (SAN) and Central Mediterranean (MEC), but including the Mediterranean region MEW into the Atlantic North (NAN, CAN).

[¥]Gene pool *k* = 2 indicates a significant split between some Atlantic regions (NAN, CAN) and the Mediterranean basin (MEW, MEC) but including the Atlantic Gulf of Cadiz region (SAN) into the Mediterranean Sea (MEW, MEC).

[»]Gene pool *k* = 3 indicates a significant split between 3 groups of regions: Atlantic North (NAN, CAN), Gulf of Cadiz (SAN) and Mediterranean Sea (MEW, MEC).

[ʈ]Gene pool *k* = 2 indicates a significant split between the Central Mediterranean region (MEC) and the rest of regions (NAN, CAN, SAN, MEW).

Table S5. Hierarchical AMOVA on Atlantic and Mediterranean hake regions and basins using nine subsets of microsatellites as classified per source (random loci and EST-diverted) as well as per selective role after LOSITAN (neutral loci *vs*. directional loci); SS, sum of squares; VC, variance component; %, percentage of variation; *F*, fixation index. * indicates that the probability of the observed value was equal or smaller than expected by random ($p \leq 0.01$); ^{ns}, non-significant *p*-value.

Hierarchical Level	Source of variation	SS	VC	%	*F*
**Microsatellite source**					
5 anonymous loci	Between basins	11.848	0.04101	1.81	$F_{CT} = 0.018^{ns\dagger}$
	Among regions within basin	16.143	0.04880	2.16	$F_{SC} = 0.022^{*}$
	Within regions	729.642	2.17240	96.03	$F_{ST} = 0.040^{*}$
21 EST-loci	Between basins	41.542	0.16266	1.79	$F_{CT} = 0.018^{*}$
	Among regions within basin	55.084	0.17940	1.97	$F_{SC} = 0.020^{*}$
	Within regions	2473.055	8.75424	96.24	$F_{ST} = 0.038^{*}$
26 loci (5 anonymous and 21 EST)	Between basins	53.390	0.20366	1.79	$F_{CT} = 0.018^{*}$
	Among regions within basin	71.227	0.22820	2.01	$F_{SC} = 0.020^{*}$
	Within regions	3202.697	10.92664	96.20	$F_{ST} = 0.038^{*}$
**Neutral microsatellites**					
4 anonymous and neutral loci	Between basins	5.342	0.00653	0.35	$F_{CT} = 0.004^{ns}$
	Among regions within basin	13.233	0.03930	2.13	$F_{SC} = 0.021^{*}$
	Within regions	610.940	1.79556	97.51	$F_{ST} = 0.025^{*}$
18 EST and neutral loci	Between basins	27.752	0.09693	1.24	$F_{CT} = 0.012^{*}$
	Among regions within basin	43.092	0.13089	1.67	$F_{SC} = 0.017^{*}$
	Within regions	2137.234	7.60016	97.09	$F_{ST} = 0.029^{*}$
22 neutral loci (4 anonymous + 18 EST)	Between basins	33.094	0.10347	1.07	$F_{CT} = 0.011^{*}$
	Among regions within basin	56.324	0.17019	1.76	$F_{SC} = 0.018^{*}$
	Within regions	2748.174	9.39573	97.17	$F_{ST} = 0.028^{*}$
**Directional microsatellites**					
1 anonymous and selective	Between basins	6.506	0.03447	8.19	$F_{CT} = 0.082^{ns\dagger}$
	Among regions within basin	2.911	0.00950	2.26	$F_{SC} = 0.025^{*}$
	Within regions	118.702	0.37683	89.55	$F_{ST} = 0.104^{*}$
3 EST and selective	Between basins	13.790	0.06572	5.18	$F_{CT} = 0.052^{ns\dagger}$
	Among regions within basin	11.992	0.04851	3.82	$F_{SC} = 0.040^{*}$
	Within regions	335.821	1.15408	90.99	$F_{ST} = 0.090^{*}$
4 selective loci (1 anonymous + 3 EST)	Between basins	20.296	0.10020	5.93	$F_{CT} = 0.059^{*}$
	Among regions within basin	14.903	0.05801	3.43	$F_{SC} = 0.037^{*}$
	Within regions	454.523	1.53091	90.63	$F_{ST} = 0.094^{*}$

†Differences in the significance of similar percentages of variation are due to the number of markers computed in each case.

Table S6. Hierarchical AMOVA on Atlantic and Mediterranean hake regions and basins using nine subsets of microsatellites as classified per origin (random loci or EST-loci) as well as per selective role after BAYESCAN (neutral polymorphism *vs*. balanced polymorphism). SS, sum of squares; VC, variance component; %, percentage of variation; *F*, fixation index. * indicates that the probabilities of the observed values were equal or smaller than expected by random ($p \leq 0.01$); ^{ns}, non-significant *p*-value.

Hierarchical Level	Source of variation	SS	VC	%	*F*
**Microsatellite source**					
5 anonymous loci	Between basins	11.848	0.04101	1.81	$F_{CT} = 0.018^{ns\dagger}$
	Among regions within basin	16.143	0.04880	2.16	$F_{SC} = 0.022^{*}$
	Within regions	729.642	2.17240	96.03	$F_{ST} = 0.040^{*}$
21 EST-loci	Between basins	41.542	0.16266	1.79	$F_{CT} = 0.018^{*}$
	Among regions within basin	55.084	0.17940	1.97	$F_{SC} = 0.020^{*}$
	Within regions	2473.055	8.75424	96.24	$F_{ST} = 0.038^{*}$
26 loci (5 anonymous and 21 EST)	Between basins	53.390	0.20366	1.79	$F_{CT} = 0.018^{*}$
	Among regions within basin	71.227	0.22820	2.01	$F_{SC} = 0.020^{*}$
	Within regions	3202.697	10.92664	96.20	$F_{ST} = 0.038^{*}$
**Neutral microsatellites**					
2 anonymous and neutral loci	Between basins	9.068	0.04414	5.08	$F_{CT} = 0.051^{ns\dagger}$
	Among regions within basin	6.412	0.02269	2.61	$F_{SC} = 0.028^{*}$
	Within regions	238.069	0.80162	92.31	$F_{ST} = 0.077^{*}$
18 EST and neutral loci	Between basins	38.213	0.15205	1.96	$F_{CT} = 0.020^{*}$
	Among regions within basin	49.517	0.17191	2.22	$F_{SC} = 0.023^{*}$
	Within regions	2027.982	7.41785	95.82	$F_{ST} = 0.042^{*}$
20 neutral (2 anonymous and 18 EST)	Between basins	47.282	0.19619	2.28	$F_{CT} = 0.023^{*}$
	Among regions within basin	55.928	0.19459	2.26	$F_{SC} = 0.023^{*}$
	Within regions	2266.050	8.21948	95.46	$F_{ST} = 0.045^{*}$
**Balanced microsatellites**					
3 anonymous and selective loci	Between basins	2.779	-0.00313	-0.22	$F_{CT} = 0.000^{ns}$
	Among regions within basin	9.732	0.02611	1.87	$F_{SC} = 0.019^{*}$
	Within regions	491.573	1.37077	98.35	$F_{ST} = 0.016^{*}$
3 EST and selective	Between basins	3.329	0.01060	0.78	$F_{CT} = 0.008^{ns}$
	Among regions within basin	5.568	0.00749	0.55	$F_{SC} = 0.006^{ns}$
	Within regions	445.073	1.33639	98.66	$F_{ST} = 0.013^{*}$
6 balanced loci	Between basins	6.108	0.00747	0.27	$F_{CT} = 0.003^{ns}$
	Among regions within basin	15.299	0.03361	1.22	$F_{SC} = 0.012^{*}$
	Within regions	936.647	2.70716	98.51	$F_{ST} = 0.015^{*}$

†Differences in the significance of similar percentages of variation are due to the number of markers computed in each case.

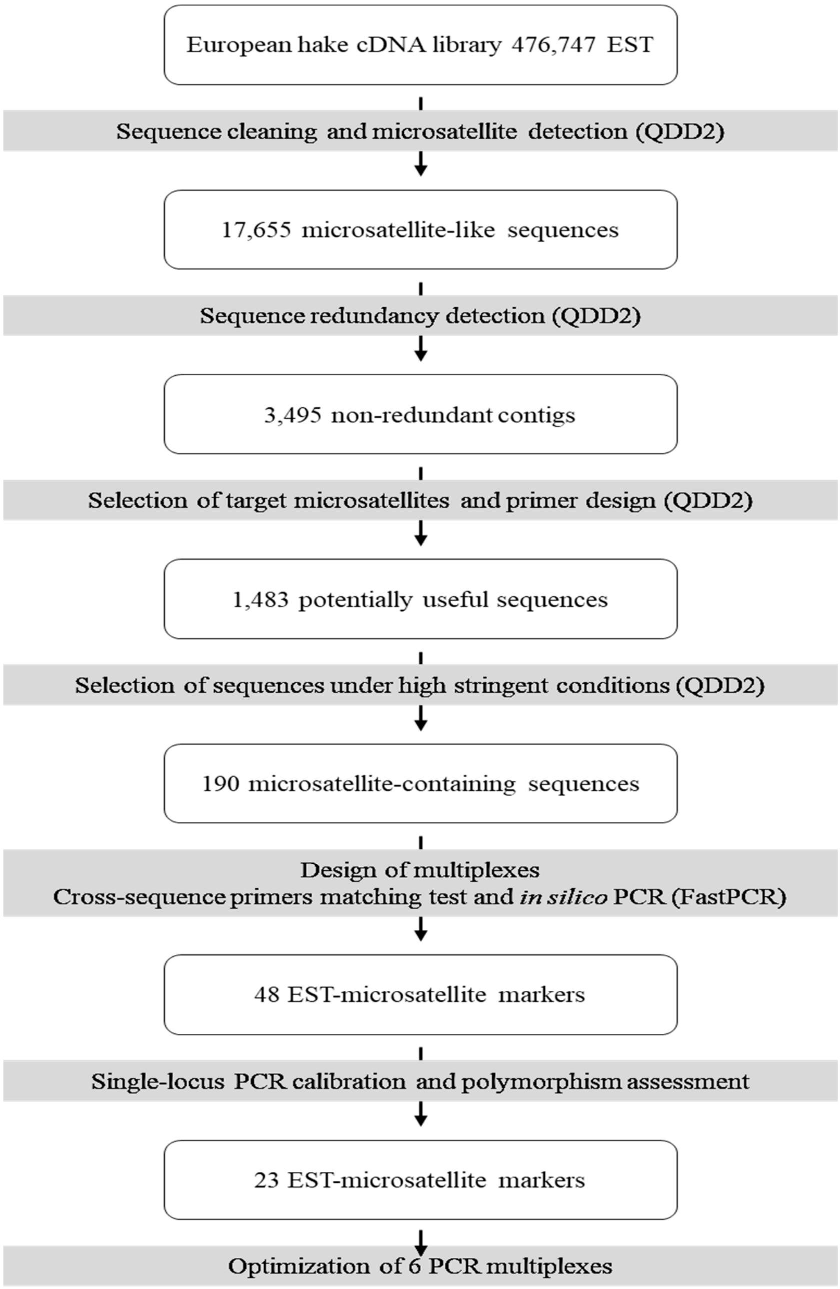

Figure S1. Roadmap for EST-microsatellite annotation from a transcriptomic cDNA library of the European hake and design of PCR multiplexes (see Table S1).
